# Repeat Dynamics across Timescales: A Perspective from Sibling Allotetraploid Marsh Orchids (*Dactylorhiza majalis* s.l.)

**DOI:** 10.1093/molbev/msac167

**Published:** 2022-07-29

**Authors:** Mimmi C Eriksson, Terezie Mandáková, Jamie McCann, Eva M Temsch, Mark W Chase, Mikael Hedrén, Hanna Weiss-Schneeweiss, Ovidiu Paun

**Affiliations:** Department of Botany and Biodiversity Research, University of Vienna, Rennweg 14, A-1030 Vienna, Austria; Vienna Graduate School of Population Genetics, Veterinärplatz 1, A-1210 Vienna, Austria; Plant Cytogenomics Research Group, CEITEC−Central−European Institute of Technology, Masaryk University, Brno 62500, Czech Republic; Central European Institute of Technology, Masaryk University, Brno 62500, Czech Republic; Institute of Experimental Biology, Faculty of Science, Masaryk University, Brno 62500, Czech Republic; Department of Botany and Biodiversity Research, University of Vienna, Rennweg 14, A-1030 Vienna, Austria; Department of Botany and Biodiversity Research, University of Vienna, Rennweg 14, A-1030 Vienna, Austria; Royal Botanic Gardens Kew, London TW9 3AE, United Kingdom; Department of Environment and Agriculture, Curtin University, Perth, Western Australia, Australia; Department of Biology, University of Lund, Sölvegatan 37, SE-223 62 Lund, Sweden; Department of Botany and Biodiversity Research, University of Vienna, Rennweg 14, A-1030 Vienna, Austria; Department of Botany and Biodiversity Research, University of Vienna, Rennweg 14, A-1030 Vienna, Austria

**Keywords:** allopolyploidy, genomic shock, genome size, marsh orchids, transposable elements

## Abstract

To provide insights into the fate of transposable elements (TEs) across timescales in a post-polyploidization context, we comparatively investigate five sibling *Dactylorhiza* allotetraploids (Orchidaceae) formed independently and sequentially between 500 and 100K generations ago by unidirectional hybridization between diploids *D. fuchsii* and *D. incarnata*. Our results first reveal that the paternal *D. incarnata* genome shows a marked increased content of LTR retrotransposons compared to the maternal species, reflected in its larger genome size and consistent with a previously hypothesized bottleneck. With regard to the allopolyploids, in the youngest *D. purpurella* both genome size and TE composition appear to be largely additive with respect to parents, whereas for polyploids of intermediate ages we uncover rampant genome expansion on a magnitude of multiple entire genomes of some plants such as *Arabidopsis*. The oldest allopolyploids in the series are not larger than the intermediate ones. A putative tandem repeat, potentially derived from a non-autonomous miniature inverted-repeat TE (MITE) drives much of the genome dynamics in the allopolyploids. The highly dynamic MITE-like element is found in higher proportions in the maternal diploid, *D. fuchsii,* but is observed to increase in copy number in both subgenomes of the allopolyploids. Altogether, the fate of repeats appears strongly regulated and therefore predictable across multiple independent allopolyploidization events in this system. Apart from the MITE-like element, we consistently document a mild genomic shock following the allopolyploidizations investigated here, which may be linked to their relatively large genome sizes, possibly associated with strong selection against further genome expansions.

## Introduction

An important evolutionary insight of recent years was the realization that whole-genome duplication (WGD) has contributed significantly to the angiosperm dominance and diversity (Soltis and Soltis 2016). Moreover, WGDs profoundly shaped the structure and function of most modern eukaryotic genomes, including many crops (Dehal and Boore 2005; Jiao et al. 2011; Van de Peer et al. 2017; Li et al. 2018). Despite their omnipresence in evolution, neopolyploids will often fail to establish (Mayrose et al. 2011) due, among other effects, to negative aspects regularly associated with their origins, including expression redundancies, regulatory incompatibilities, and epigenetic imbalances that can activate transposable elements (TEs) (Ramsey and Schemske 2002; Chen 2007; Paun et al. 2007; Doyle et al. 2008; Parisod et al. 2010). Such challenges have been together referred to as a “genomic shock” (McClintock 1984), which is likely to be especially profound in early-generation allopolyploids that combine two divergent parental genomes. Allopolyploidization is therefore expected to initiate a plethora of stochastic, cascading molecular responses with immediate implications for the adaptive success of neoallopolyploids.

In the long term, WGD is followed by diploidization, a repeating process that results in polyploidization–diploidization cycles (Van de Peer et al. 2009; Jiao et al. 2011; Wendel 2015; Chase et al. 2022). When distinct genomes are combined together, one of them can dominate the other during the diploidization process to ultimately result in “biased fractionation” (Sankoff et al. 2012). This model explains allopolyploid genome evolution starting from epigenetic differences linked to an imbalance between parental insertion load of TEs: the smaller subgenome with a lower TE load becoming dominant in the resulting polyploid and therefore leading to different levels of heterochromatinization between homoeologs (Garsmeur et al. 2014; Woodhouse et al. 2014). These differences trigger unequal expression patterns between parental subgenomes, eventually resulting in preferential degradation of one of the homoeologs (Cheng et al. 2016; Edger et al. 2017; Wendel et al. 2018; Gaebelein et al. 2019; Mhiri et al. 2019; Eriksson et al. 2020). Such a process has been reported to be initiated for example in the neoallopolyploid *Mimulus peregrinus,* strongly associated with subgenome-wide methylation levels and homeolog expression bias (Edger et al. 2017).

On the other hand, the “nuclear-cytoplasmic interaction” hypothesis (Song et al. 1995; Leitch et al. 2006; Dodsworth et al. 2020) proposes that the maternal subgenome of an allopolyploid will become dominant over the paternal subgenome due to potential incompatibilities between the maternally inherited cytoplasmic environment and the paternal subgenome. In turn, this incompatibility would lead to asymmetric expression, retention and degradation between the subgenomes as a function of the degree of divergence between the diploid parental species (Song et al. 1995). With regard to mobile elements, the maternal subgenome of allopolyploids is also initially expected to be more efficient in controlling its TEs based on maternally inherited cytoplasmic TE-repressing factors, such as small interfering RNAs (Vicient and Casacuberta 2017). For example, a bias towards paternal subgenome degradation has been reported for *Nicotiana tabacum* (Leitch et al. 2006; Renny-Byfield et al. 2011). However, in several other study systems this pattern was clearly not detected (e.g., Parisod et al. 2009; Mhri et al. 2019; Dodsworth et al. 2020). Nonetheless, altogether post-WGD TE activation is often (Kashkush et al. 2003; Parisod et al. 2010; Renny-Byfield et al. 2011; Oliver et al. 2013; Vicient and Casacuberta 2017; Chase et al. 2022), but not always (IWGSC et al. 2018; Burns et al. 2021) regarded as a key driver of adaptation to the polyploid state and of further evolution.

In addition to relatively continuous low-frequency transposition, TEs proliferate in bursts driven by genomic (Parisod et al. 2010; Wendel et al. 2018) or environmental disturbances (Chuong et al. 2017; Dubin et al. 2018), often coupled with demographic factors that impair efficient selection. As each burst is expected to be a largely independent, erratic event, TE mobilization may vary widely across populations and evolutionary stages. It has been shown that the composition and copy number of TEs can differ significantly between closely related species, which may otherwise display general synteny (Devos 2005; Willing et al. 2015), even between and within conspecific populations (Springer et al. 2016; Carpentier et al. 2019). This variation can translate into phenotypic diversity (Parisod et al. 2009; Sigman and Slotkin 2016; Springer et al. 2016; Chuong et al. 2017; Vicient and Casacuberta 2017; Dubin et al. 2018; Weissensteiner et al. 2020) when TEs affect in *cis* the activity of adjacent genes, for example by disrupting open reading frames when inserting in exons, acting as regulatory elements when inserting in promoters or introns, triggering transcript truncation and other novel splicing variants, or attracting epigenetic marks to the respective genomic region, thereby down-regulating or silencing genes. Such phenotypic effects will be immediately visible to selection. In recurrently formed neoallopolyploids, the content, positions and activity of TEs can therefore influence divergent trait expression, potentially leading to distinct phenotypes and environmental trajectories that may aid establishment as viable species, independent of diploid and polyploid relatives (Lynch and Force 2000; Soltis et al. 2004; Comai 2005; Jackson and Chen 2010). However, it is still unclear in which conditions repeated allopolyploidization will ultimately result in species with different evolutionary trajectories (Soltis et al. 2010) and how such sibling species maintain distinctiveness despite sharing the same ploidy and genetic background.

**Fig. 1.**
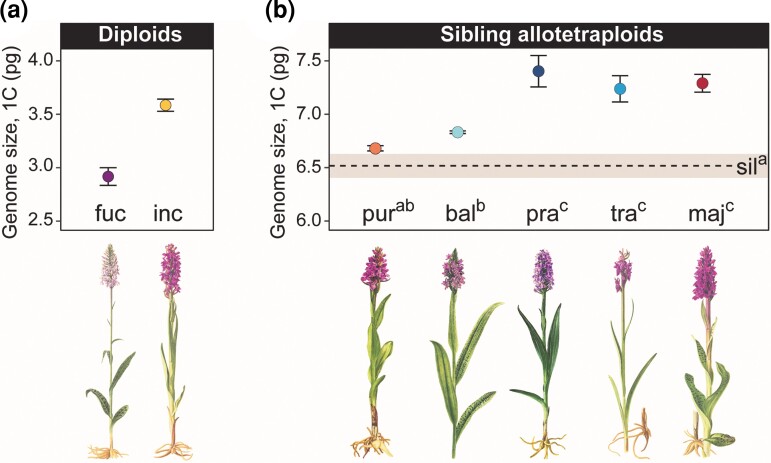
The *Dactylorhiza* species investigated here and their genome sizes as estimated for this study, given as 1C values in picograms (pg) plus error bars as standard error of the mean. Note the difference genome-size scale on the Y-axes in (*a*) and (*b*). (*a*) Diploids: fuc—*D. fuchsii*, inc—*D. incarnata*. (*b*) Allotetraploids: bal—*D. baltica*, maj—*D. majalis*, pra—*D. praetermissa*, pur—*D. purpurella*, sil—*in silico* allopolyploid, tra—*D. traunsteineri*. The dashed line in (*b*) represents the mean of the additive genome size of the diploid parents (*D. fuchsii* and *D. incarnata*), whereas the grey area shows the 95% confidence interval. Superscript letters (^a^/^b^/^c^) are used to display significant differences in genome size. Absence of shared letters (e.g., ^ab^ vs. ^c^) indicates a significant difference in genome size (supplementary table S2, Supplementary Material online). Ordering of the allotetraploids on the X-axis follows the relative age estimates according to Brandrud et al. (2020), with *D. purpurella* further estimated to be *ca* 530 generations old and *D. majalis ca* 104,000 generations (maximum estimates from Hawranek 2021). Plant illustrations modified from Nelson (1976).

In this paper, we aim to provide insights into the fate of TEs across early stages of allopolyploid evolution by focusing on a naturally occurring series of independently established sibling allopolyploid marsh orchids (*Dactylorhiza majalis* s.l., Orchidaceae). We study five sequentially produced allotetraploids, *Dactylorhiza baltica, D. majalis* s.str., *D. praetermissa, D. purpurella* and *D. traunsteineri* s.l. (fig. 1) (Devos et al. 2006; Pillon et al. 2007; Brandrud et al. 2020), and their diploid parents, *D. fuchsii* and *D. incarnata*. Numerous previous studies have established that diploids in the *Dactylorhiza* genus have 20 chromosome pairs (2*n* = 2*x* = 40), whereas allotetraploids have 40 chromosome pairs (2*n* = 4*x* = 80) (Hagerup 1938; Vermeulen 1938; Heslop-Harrison 1953; Holmen and Kaad 1956; Lord and Richards 1977; Jonsell 1982; Lövkvist and Hultgård 1999). Since the divergence of the two diploid parental species around 5.5 MYA (Inda et al. 2012; Brandrud et al. 2020), their genomes have diverged under different demographic dynamics. In contrast to *D. fuchsii*, *D. incarnata* apparently suffered a dramatic bottleneck, likely related to a long-distance dispersal event from Asia to Europe roughly 1.5 MYA (Hedrén 1996; Pillon et al. 2007; Balao et al. 2016, 2017; Brandrud et al. 2020). Related to such a relatively recent bottleneck, Balao et al. (2017) have provided evidence for an overexpression in *D. incarnata* relative to *D. fuchsii* of the RNA-dependent DNA polymerase (RdDp) pathway and DNA integration, likely associated with transposition of mobile elements. These results, together with the divergent genome sizes of the diploids (1C-value estimates of 2.89 pg for *D. fuchsii*, and 3.55 pg for *D. incarnata*; Aagaard et al. 2005) suggest a distinct repeat load in the diploids, potentially driving significant genomic conflicts and dramatic genomic shock in their hybrids. Indeed, despite hybridizing on several occasions, these diploids have not produced any homoploid hybrid species (Pillon et al. 2007; Paun et al. 2009; Brandrud et al. 2020). However, the TE landscape of the two diploid parents and allopolyploids has not been previously studied.

Recent genomic work based on RAD-seq across the orchid genus *Dactylorhiza* (Brandrud et al. 2020) illustrated repeated, unidirectional, independent allopolyploidization involving the two diploid parents, resulting in an array of ecologically divergent, sibling allotetraploids (Hedrén 1996; Dijk and Grootjans 1998; Pillon et al. 2007; Hedrén et al. 2011; Paun et al. 2011; Wolfe et al. 2021). Based on the number of private alleles accumulated in each allopolyploid, Brandrud et al. (2020) inferred relative ages for these allopolyploidizations, later confirmed by Hawranek (2021) that estimated with coalescent methods maximum ages for the allopolyploids ranging from *ca*. 530 generations for *D. purpurella*, to ca. 74,000 generations for *D. traunsteineri* and *ca*. 104,000 generations for *D. majalis* s.str. Making use of this series of sibling allopolyploid marsh orchids of different ages, we specifically ask: 1) does WGD trigger monoploid genome size changes in *Dactylorhiza majalis* s.l. and what are the temporal dynamics of these changes, 2) which TE families experience changes post-polyploidization and does this relate to imbalances between parental TE loads, and 3) are the observed large-scale changes repeated among the sibling allopolyploids?

## Results

### Genome Size of Early Allopolyploids Is Consistent with Parental Additivity, But Those of Older Allotetraploids Are Larger Than Expected

To allow comparative analyses of repeat composition in the sibling *Dactylorhiza* allopolyploids and their diploid progenitors, we first estimated genome sizes for multiple accessions of each species (supplementary table S1, Supplementary Material online) using flow cytometry. For the diploid parental species, we obtained 1C genome sizes consistent with previous estimates (Aagaard et al. 2005): 2.93 pg versus 2.89 pg for *D. fuchsii*, and 3.60 pg versus 3.55 pg for *D. incarnata* (fig. 1*a*, table 1; supplementary table S1, Supplementary Material online).

**Table 1.** Flow Cytometry Estimates of Genome Size.

Species	Ploidy	N^a^	1C (pg)^b^	1C, SEM^c^	2C (Gbp)^b^
*Dactylorhiza fuchsii*	2x	4	2.93	±0.18	5.73
*D. incarnata*	2x	7	3.60	±0.14	7.04
*D. purpurella*	4x	5	6.69	±0.05	13.09
*D. baltica*	4x	7	6.83	±0.03	13.37
*D. praetermissa*	4x	5	7.40	±0.29	14.47
*D. traunsteineri*	4x	5	7.24	±0.25	14.16
*D. majalis*	4x	8	7.28	±0.16	14.24

Individual genome-size measurements are reported in supplementary table S1, Supplementary material online.

a N, sample size.

b The mean genome size, given as picograms (1C) or giga base pair (2C).

c SEM, standard error of the mean.

Genome sizes for the sibling allopolyploids varied between 6.69 pg/1C for *D. purpurella* and 7.40 pg/1C for *D. praetermissa* (fig. 1*b* and table 1). Comparison of the observed and expected (i.e., additive relative to the parents) genome sizes of allotetraploids revealed that the youngest allopolyploid, *D. purpurella,* previously estimated to be maximum 530 generations old (Hawranek 2021) fell closest to but slightly outside the expected value (fig. 1*b*). The significance of differences in genome sizes between pairs of species and between each allotetraploid and additive expectations is indicated in figure 1 and in supplementary table S2, Supplementary Material online. Taking into account relative (Brandrud et al. 2020) and absolute age estimates (Hawranek 2021) to order the allotetraploid formations through time, our results (fig. 1*b*, and table 1) therefore indicate that the allotetraploid genome size is initially additive, but appears enlarged by *ca.* 13.5% in comparison to expectations in intermediate-aged and older allopolyploids (Hawranek 2021).

### The Diploid Parental Species have a Distinct Repeat Composition

**Fig. 2.**
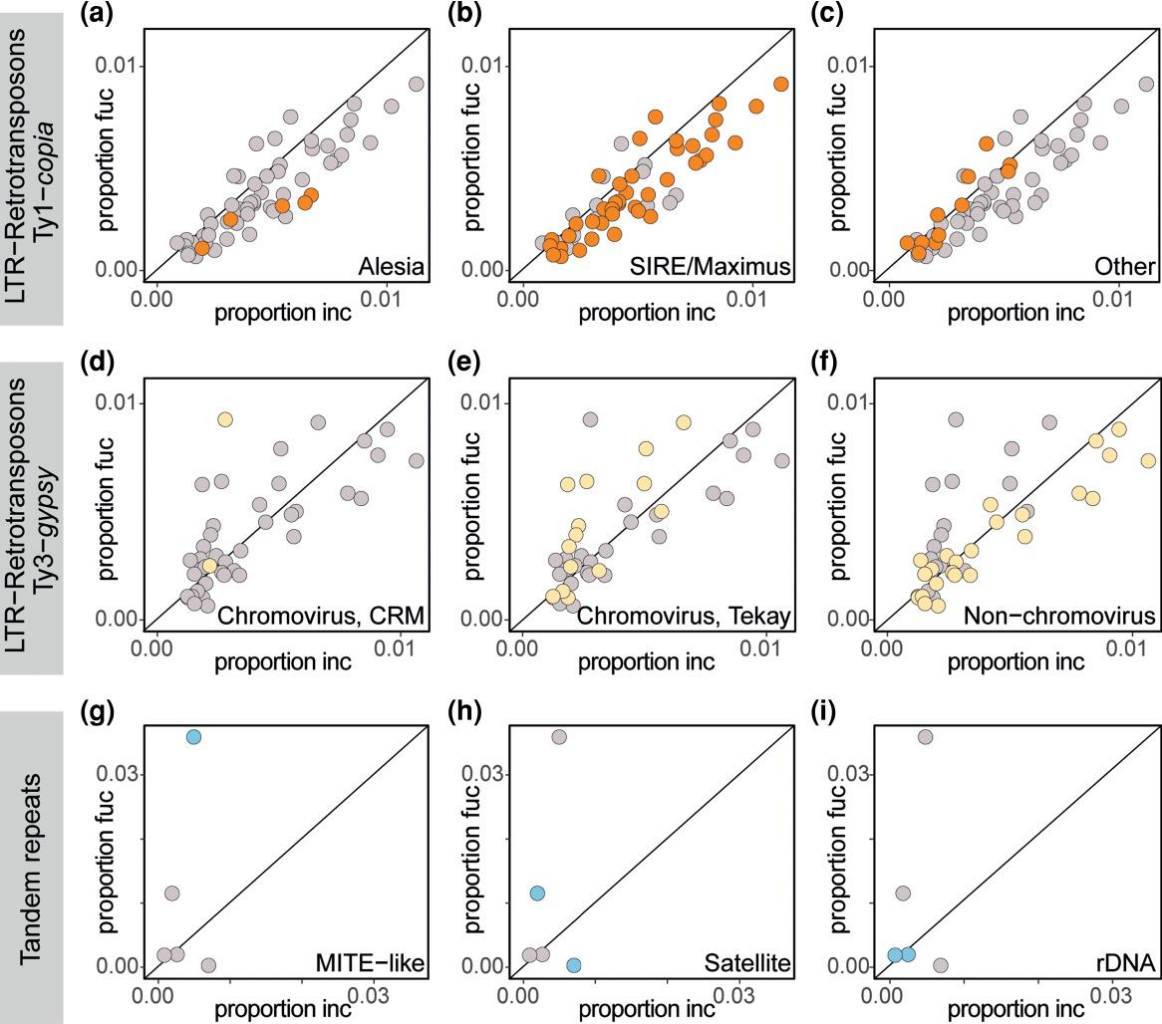
Repeat comparison for the parental diploid species. Cluster proportion for different repeat classes in paternal *D. incarnata* (“inc”, X-axis) against the maternal *D. fuchsii* (“fuc”, Y-axis). Panels (*a*–c) show LTR-retrotransposons Ty3-*gypsy* elements in grey, with specific families highlighted in yellow: chromovirus, subfamily CRM (*a*); chromovirus, subfamily Tekay (*b*); non-chromovirus (*c*). Panels (*d*–*f*) show LTR-retrotransposons Ty1-*copia* elements in grey, with specific families highlighted in orange: Alesia (*d*); SIRE/Maximus (*e*); other Ty1-*copia* families (*f*). Panels (*g–i*) show tandem repeat clusters in grey, with specific classes highlighted in blue: MITE-like element (*g*); satellite DNA (*h*); 5S and 35S rDNA (*I*).

To assess the relative repeat composition of the two parental diploids and their allotetraploids, we used Illumina whole-genome skimming for 35 accessions representing seven species (supplementary table S4, Supplementary Material online) and the RepeatExplorer pipeline (Novak et al. 2013; Novak et al. 2020b). When comparing the abundances (supplementary table S3, Supplementary Material online) and relative proportions (table 2) of tandem and dispersed repeat proportions between the two parental diploids, our results confirmed that *D. incarnata* has overall higher repeat content corresponding to its larger genome (total repeats estimated at 2.6 Gb for *D. incarnata* vs. 2.03 Gb for *D. fuchsii*, calculated on haploid genome sizes; supplementary table S3, Supplementary Material online). When looking at the different repeat types, we found that Ty1-*copia* LTR-retrotransposons are in general more abundant in the *D. incarnata* genome in comparison to *D. fuchsii* (1.75 Gb vs. 1.28 Gb, calculated on haploid genome sizes; supplementary table S2, Supplementary Material online; fig. 2*a–c* and table 2) and appear to drive most of the genome size difference between genomes of the two diploid parents (fig. 1). Ty3-*gypsy* LTR-retrotransposons exhibit a more heterogeneous pattern for the two diploid genomes (fig. 2*d–f*, table 2; supplementary table S3, Supplementary Material online) with chromoviruses being more abundant in the genome of *D. fuchsii* and non-chromoviruses in the *D. incarnata* genome. Lastly, we found one element, a putative tandem repeat, potentially derived from a non-autonomous miniature inverted-repeat TE (MITE), that makes up a difference of more than 170 Mbp between the two diploids and is more abundant in the smaller genome of *D. fuchsii* (i.e., the MITE-like element makes up 206.3 Mb in the diploid genome size of *D. fuchsii* and 35.2 Mb for *D. incarnata*; see fig. 2*g*, table 2; supplementary table S2, Supplementary Material online). Finally, we identified two potentially differentiated satDNAs in each diploid (fig. 2*h*), but little difference in the proportion of clusters annotated as rDNAs (fig. 2*i*).

**Table 2.** Genome Proportion Estimates (%) of Repeats for Each Species Analysed Here.

Repeat type/family	fuc	inc	pur	bal	pra	tra	maj
LTR retrotransposons	44.8	49.6	48.2	47.0	45.7	45.9	46.0
Ty1-copia	22.9	27.7	26.2	26.0	24.8	25.0	24.8
SIRE/maximus	17.0	21.2	19.7	19.8	18.8	18.7	18.4
Other	5.9	6.6	6.5	6.2	6.0	6.3	6.5
Ty3-gypsy	17.5	16.5	16.8	16.1	16.0	16.1	16.2
Chromovirus	8.1	5.5	6.6	6.3	6.4	6.3	6.4
CRM	1.4	0.7	1.0	1.0	1.0	0.9	1.0
Tekay	6.3	4.5	5.2	5.0	5.1	5.0	5.0
Other	0.4	0.3	0.4	0.4	0.4	0.4	0.4
Non-chromovirus	9.4	11.0	10.3	9.8	9.6	9.8	9.8
Athila	3.2	4.6	4.0	3.9	3.8	3.8	3.7
Retand	6.2	6.4	6.3	5.9	5.8	6.0	6.1
LTR unclassified	4.4	5.4	5.2	5.0	4.9	4.9	5.0
Non-LTR retrotransposons	0.4	0.4	0.4	0.4	0.4	0.4	0.4
LINE	0.4	0.4	0.4	0.4	0.4	0.4	0.4
DNA transposons	0.4	0.4	0.4	0.4	0.4	0.4	0.4
hAT	0.3	0.3	0.3	0.3	0.2	0.3	0.3
MITE	0.06	0.06	0.06	0.05	0.05	0.05	0.06
Other	0.1	0.1	0.1	0.1	0.1	0.1	0.1
Tandem repeats	5.2	1.9	2.5	4.6	7.9	5.8	5.8
rDNA	0.4	0.4	0.3	0.3	0.2	0.2	0.3
satDNA	4.8	1.5	2.2	4.3	7.7	5.6	5.5
MITE-like	3.6	0.5	1.0	3.2	6.6	4.5	4.5
Unclassified	7.3	8.6	8.3	8.0	7.8	7.9	8.1
Total TEs	45.6	50.4	48.9	47.8	46.5	46.7	46.9
Total repeats	71.0	73.8	72.1	73.3	73.8	73.0	72.6

Species abbreviations are explained in the legend of figure 1.

### Net Repeat Content Increases with Age among the Sibling Allopolyploids up to a Certain Level

**Fig. 3.**
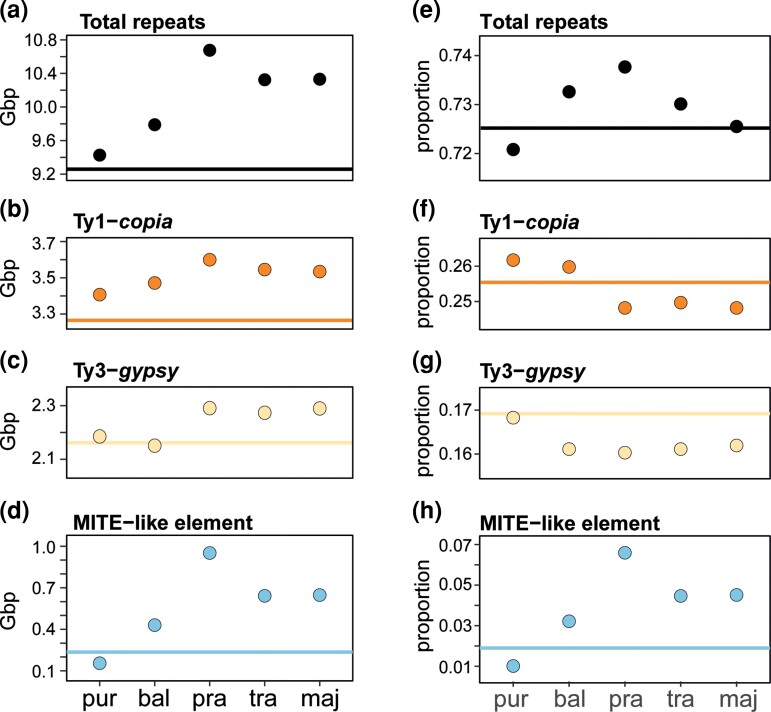
Repeat composition in Gbp *(a–d)* and relative proportions of repeats classifications (*e–h*) for the sibling allotetraploid *Dactylorhiza* analysed here. Species abbreviations follow figure 1. The lines indicate the expected additivity of the parental diploids (*D. fuchsii* and *D. incarnata*) for the respective type of elements. Ordering of the allotetraploids on the X-axis follows the relative age estimates according to Brandrud et al. (2020), with *D. purpurella* further estimated to be *ca*. 530 generations old and *D. majalis ca*. 104,000 generations (maximum estimates from Hawranek 2021). Note the magnified scale of the Y-axes.

The proportion estimates of total repeats for the allotetraploids were found to range from 72.1% of the genome for *D. purpurella* up to 73.8% for *D. praetermissa* (table 2 and fig. 3*e*). These proportions were similar to the additive expectation of 72.5%. The pattern of total repeats (fig. 3*a*; supplementary table S3, Supplementary Material online) showed a maximum net difference of almost 1.25 Gbp between *D. purpurella* and *D. praetermissa*, corresponding well to the maximum observed difference in genome size estimated by flow cytometry (i.e., 1.38 Gbp between the same species; table 1). With respect to the previously estimated age variation between the allotetraploids (Brandrud et al. 2020; Hawranek 2021), the lowest estimate for total repeats was recovered in the youngest allotetraploid, *D. purpurella*—an estimate slightly higher than expected (fig. 3*a*; supplementary table S3, Supplementary Material online). The genome length representing the repeats is markedly increased compared to expectations for the intermediate aged (i.e., *D. praetermissa* and *D. traunstenieri*) and the oldest allotetraploids (i.e., *D, majalis*), but it is not remarkably different between them.

Over 45% of the allotetraploid genomes was found to be represented by LTR retrotransposons (table 2), mainly Ty1-*copia* (ranging among allotetraploids between 24.8 and 26.2%) and Ty3-*gypsy* (between 16 and 16.8%; supplementary fig. S1*b*and*c*, Supplementary Material online). In both Ty1-*copia* and Ty3-*gypsy* families an increase in net amounts is estimated, in particular in the older allotetraploids (fig. 3*b*and*c*; supplementary table S3, Supplementary Material online). However, in genomic proportions we observe slightly less than parental additivity for LTRs in most allotetraploids (fig. 3*f*and*g*andtable 2). Both Ty1-*copia* and Ty3-*gypsy* elements are abundant in these polyploid genomes (fig. 3*b*and*c*; supplementary table S3, Supplementary Material online), and the differences in LTR elements between the young and old allotetraploids contribute altogether ca. 300 Mb to the maximum observed variation in genome size.

**Fig. 4.**
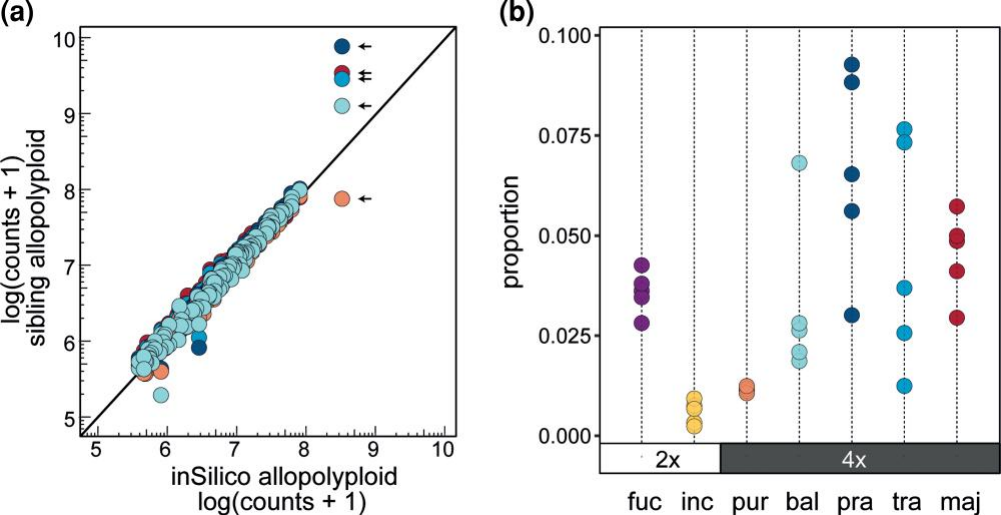
Repeat comparison for sibling allotetraploids. (*a*) The relative proportion of repeat elements of each allotetraploid (Y-axis) relative to an *in silico* allopolyploid (X-axis), representing the expected additivity. The repeat clusters for each allotetraploid are shown in colors corresponding to those used in panel (*b*). A MITE-like element is indicated with arrows. (*b*) Within-species variation of the MITE-like element. Ordering of the allopolyploids on the X-axis follows age estimates following Brandrud et al. (2020) and Hawranek (2021). Species abbreviations follow figure 1.

**Fig. 5.**
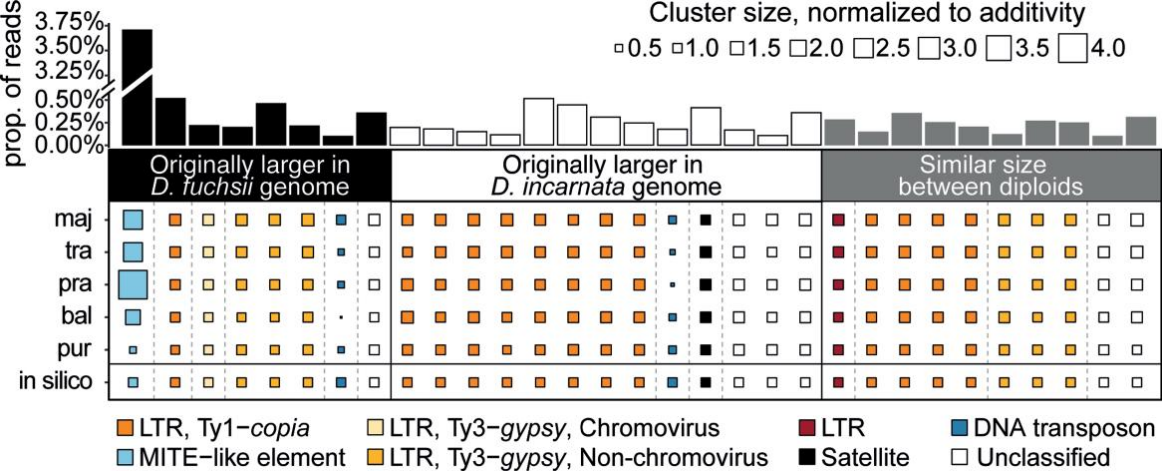
Repeat elements with at least 20% size difference between the observed value of at least one allopolyploid and the expected (*in silico*). The clusters are sorted into groups originally larger in a parental species or similar in size. Each column of the square plot is normalized by the expected value (*in silico*), with smaller squares indicating a decrease whereas larger squares indicate an increase in size according to the legend. The color of the squares represents repeat types. The bar graph on top shows the size of a cluster in the analysis, that is, the proportion of reads out of all analysed reads (note the break in the Y scale going from 0.50 and 3.25%).

**Fig. 6.**
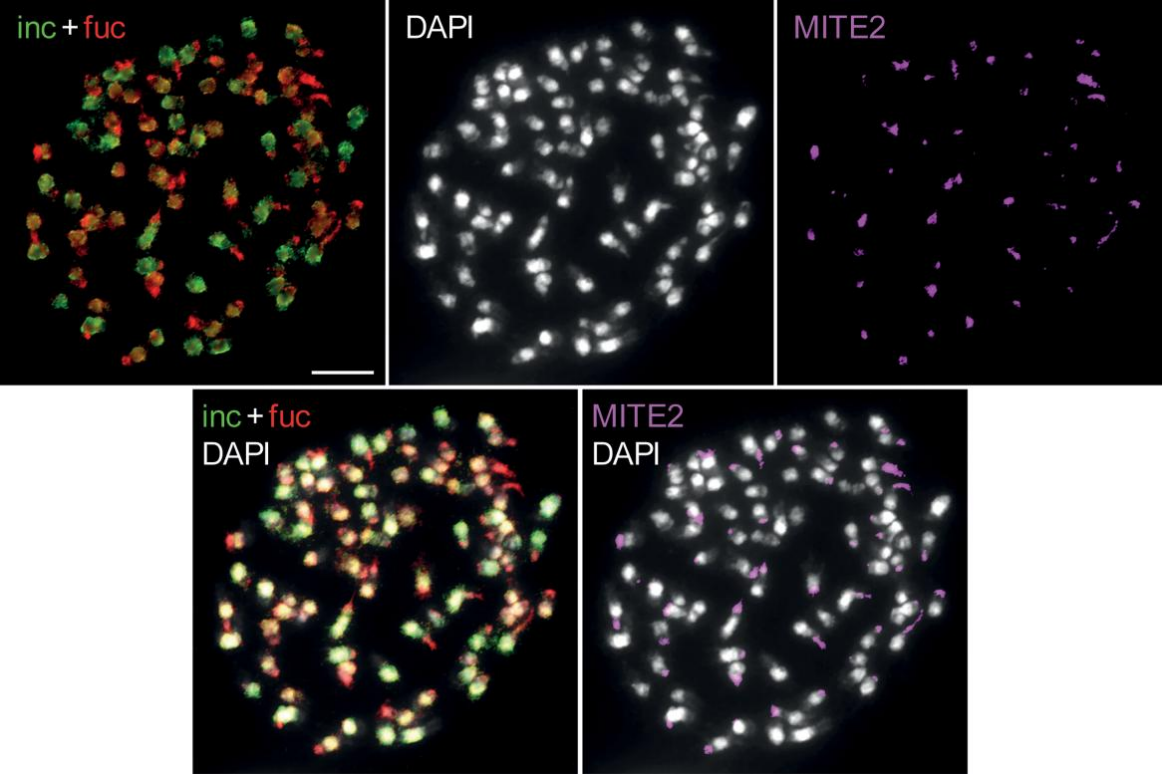
GISH in *D. majalis* labelled with genomic DNA of *D. incarnata* and *D. fuchsii* as probes, followed by FISH localization of the MITE-like repeat. Chromosomes were counterstained by DAPI; GISH and FISH signals are shown in color as indicated. Scale bar, 10 µm.

More than 800 Mbp of the maximum difference observed among allotetraploid genome sizes appears to be driven by tandem repeats (supplementary table S3, Supplementary Material online), which are found in genomic proportions ranging between 2.5% for *D. purpurella* and 7.9% for *D. praetermissa* (table 2). In particular, a specific element, potentially derived from a MITE, which shows variation between the diploid parental representatives (with larger amounts in the maternal species, *D. fuchsii*; fig. 2*g*), is found to be highly variable among the allotetraploid species (fig. 3*d*, fig. 4*a*, table 2; supplementary table S2, Supplementary Material online), but also between individuals within all allotetraploids, except the youngest *D. purpurella* (fig. 4*b*). This represents the largest cluster in the analysis (fig. 5), as well as the element contributing the most to the observed genome size difference among the allotetraploids. The cluster localizes to subterminal chromosomal positions (see below and fig. 6) and may represent a putative tandem repeat, although it still carries genomic signatures of MITEs, specifically inverted repeats that may represent original TIRs. Therefore, we refer to it here as a MITE-like element.

To investigate a potential subgenome dominance bias in the allotetraploids, we compared the observed estimates in the sibling allopolyploids to an expected value of an *in silico* allopolyploid to extract clusters different in size from the expected. We further classified the respective clusters as originally larger (i.e., by at least 20%) in a parental species or similar in size between the parents. The largest observed difference was the change in the MITE-like element (fig. 5). We also found that among the clusters originally larger in the paternal *D. incarnata* genome mainly Ty1-*copia* elements were different among the allopolyploids, whereas among the clusters originally larger in the maternal *D. fuchsii* mainly Ty3-*gypsy* elements were different (fig. 5).

We then looked into specific results for different TE families. We normalized each cluster relative to the *in silico* allopolyploid with values between −1 and 1, where a value of 0 means that the cluster equals the *in silico* allopolyploid and by extension the expectation from the parental species. We found a few elements with large effects scattered among families (supplementary fig. S2, Supplementary Material online).

### The Highly Dynamic MITE-Like Element Localizes in Subterminal Positions on Most Allotetraploid Chromosomes

Due to the highly dynamic amounts recovered for this MITE-like element, we further used a combination of genomic *in situ* hybridization (GISH) and fluorescence *in situ* hybridization (FISH) in accessions of *D. majalis* and *D. traunsteineri* to investigate its location in the allotetraploid genomes and establish if it is interspersed or clustered. This MITE-like element was localized in subterminal chromosomal positions on nearly all chromosomes of the allotetraploids (fig. 6; supplementary fig. S3, Supplementary Material online). Because we estimated with RepeatExplorer a significant difference in the amount of the MITE-like element copies in the parental diploids (fig. 2*g*, fig. 4*b*, table 2; supplementary Table S3, Supplementary Material online), we confirmed with FISH its relatively high copy number in the maternal diploid, *D. fuchsii* (supplementary fig. S3*b*, Supplementary Material online), in contrast to *D. incarnata* that showed no detectable signal (supplementary fig. S3*a*, Supplementary Material online). We were further interested if the MITE-like element has spread among the two subgenomes of the allotetraploids. Therefore we stained the two subgenomes and the MITE-like element with fluorescent dyes and observed that this element is found in both, but it provides comparatively larger signals on the *D. fuchsii* allotetraploid subgenomes (fig. 6).

## Discussion

### Rampant TE Amplification Is Observed Only in Older Allopolyploids

Genome reorganizations after allopolyploidization have been investigated in different systems, from recent neoallopolyploids (e.g., 140 years old *Mimulus peregrinus*, Edger et al. 2017) to old, well-established paleopolyploids (e.g., ∼2–4 Ma tetraploid and hexaploid *Spartina*, Giraud et al. 2020, and ∼6 Ma *Nicotiana* sect. *Suaveolentes*, Dodsworth et al. 2020; Chase et al. 2022), and from synthetic allopolyploids (e.g., *Nicotiana*, Mhiri et al. 2019) to natural populations (e.g., *Spartina anglica*, Parisod et al. 2009). However, to our knowledge, mobile element turnover after allopolyploidization has not been evaluated thus far in multiple allopolyploid species formed independently and unidirectionally by the same parental diploids. The sibling allotetraploid marsh orchids studied here fill in an important knowledge gap because they allow testing if these replicated the events following genome merger. Allotetraploids in *Dactylorhiza majalis* s.l. are still in the early phases of their evolution, but they are established species (Pillon et al. 2007; Brandrud et al. 2020; Hawranek 2021), with large distributions and distinct ecologies (Hedrén et al. 2011; Paun et al. 2011; Wolfe et al. 2021), and therefore represent an excellent model for studying post-WGD processes through time.

In the youngest of the sibling marsh orchids studied here, *D. purpurella* (maximum 530 generations old, Hawranek 2021; see also Hedrén et al. 2011, Brandrud et al. 2020) we observe nearly the expected (additive) genome size (fig. 1) and repeat content (fig. 3). Previous studies of rDNA ITS sequences have also shown that *D. purpurella* individuals often exhibit an equal proportion of the parental alleles (Pillon et al. 2007). These observations are consistent with findings in other systems, for example in synthetic allopolyploid wheat (Kashkush et al. 2003), *Brassica napus* (Sarilar et al. 2013) and *Nicotiana* (Mhri et al. 2019), where no or only minimal transposition and gene conversion have been reported after recent genome merger. The situation is different in *Tragopogon* neoallotetraploids, where homogenization of rDNA repeats happens quickly and in alternate directions in different populations (Kovarik et al. 2005; Matyášek et al. 2007). In resynthesized *Mimulus perregrinus*, Edger et al. (2017) reported reduced levels of CHH methylation within TEs that should promote their activation, and Kashkush et al. (2003) documented in synthetic wheat transcriptional upregulation of some retrotransposons despite a lack of net TE amplification. However, repressive control of retroelements seems often to be maintained in early stages of allopolyploid evolution, perhaps at the level of post-transcriptional regulation via heterochromatic small interfering RNAs (Wendel et al. 2016). We find this phase in which allopolyploids maintain a largely additive genome size to extend at least for hundreds of generations after whole genome doubling in marsh orchids, but in other systems it may cover tens to hundreds of thousand generations, as reported for example for *Arabidopsis suecica* (Burns et al. 2021); *Melampodium strigosum* (McCann et al. 2018) and *Nicotiana rustica* (Dodsworth et al. 2020).

However, in the case of *Dactylorhiza*, in species between hundreds and thousands of generations post-allopolyploidization (i.e., for *D. baltica* and *D. praetermissa*), we observe a genome upsizing close to 1 Gbp (1C) (fig. 1) and a corresponding level of repeat amplification (fig. 3). The allopolyploids could have experienced a phase when drift rather than selection affected their genomes as they likely expanded the sizes of their populations, niches and distribution. Repeat amplification in marsh orchids seems to involve largely proportional changes in copy numbers across the majority of repeat families, both tandem and dispersed, but it is particularly driven by changes in a MITE-like element. A correlation between genome size and number of TEs in a genome is widely accepted (Kidwell 2002; Touchon and Rocha 2007; Elliott and Gregory 2015). However, a limit to this correlation appears in the context of allopolyploid evolution in *Dactylorhiza*, as due to amplification of the MITE-like element, the proportion of other types of repeats in the genome appears to slightly decrease (fig. 3*f*and*g* and table 2) even though the net number of repeats increases (fig. 3*b*and*c*; supplementary table S3, Supplementary Material online) (Kelly et al. 2015; Novak et al. 2020a). In particular, in *D. praetermissa*, which must have had fewer than 74,000 generations (Brandrud et al. 2020; Hawranek 2021), total repeat number appears to have expanded with 14.5% relative to expectations (fig. 3*a*). Such an extensive TE mobilization may be associated with significant chromosomal rearrangements and impact evolution of genes and functions (Wendel et al. 2018). Similar but even more extensive TE amplification of around 29% was also observed in *Nicotiana repanda*, estimated to be roughly 4 M generations old (Dodsworth et al. 2020), much older than these allopolyploid marsh orchids.

Finally, no significant large scale differences are obvious between *Dactylorhiza traunsteineri* and *D. majalis* (fig. 3), which are each likely tens of thousands generations old (their origins predate the last glacial maximum; Hawranek 2021), and occur widely across Europe (Wolfe et al. 2021). Concerted evolution of rDNA alleles is well underway in these allopolyploids (Pillon et al. 2007), with *D. majalis* typically exhibiting only maternal copies. In multiple other established species a decrease of TE content rather than an increase compared to expected was evident over the long term (e.g., Ozkan et al. 2003; Leitch and Bennett 2004; Eilam et al. 2009; Chase et al. 2022). Indeed, it is generally accepted that over broad evolutionary time scales polyploidy is not a major factor in genome size increase (Wang et al. 2021; Chase et al. 2022). Our results confirm that the mechanisms controlling genome expansion and downsizing post-WGD obviously act on different time horizons. A similar age effect has been observed previously in *Nicotiana*, albeit across species that did not fully share parentage (Dodsworth et al. 2020). Our findings follow a similar pattern across timescales as in *Nicotiana*, although at a smaller scale and without a significant decrease compared to additivity; possibly our oldest allotetraploid, *D. majalis*, is too young (∼100k generations; Hawranek 2021) to show significant genome downsizing.

### Element-Specific, But No Genome-Wide Parental Bias in TE Dynamics

Allopolyploidization can trigger genomic shock (McClintock 1984), imbalancing epigenetic control of TEs that are thus prone to undergo an acute proliferation (Wendel et al. 2016). In early stages of allopolyploid evolution, selection is also likely reduced, whereas the highly duplicated genomic landscape will mask deleterious effects of individual TE insertion in or around genes (Comai 2005). Our results document divergent genomic compositions in the parental diploid species, *D. incarnata* and *D. fuchsii* (fig. 2), and confirm a more than 20% difference between genome sizes (fig. 1*a*), suggesting a high potential for genomic shock in the resulting allotetraploids. In stark contrast to this expectation, we consistently observe that in the sibling *Dactylorhiza* allotetraploids nearly all TEs conform to the additive expectations (fig. 4*a*; supplementary fig. S1, Supplementary Material online), which has also been reported in other allopolyploids (e.g., McCann et al. 2018; Mhiri et al. 2019; Chen ZJ et al. 2020; Dodsworth et al. 2020; Burns et al. 2021). An overall genomic increase of TEs compared to expectation was also not found in *Capsella bursa-pastoris* (Ågren et al. 2016), but an increase in TE abundance in gene-rich regions was documented, consistent with the hypothesis that relaxed selection rather than an epigenetic imbalance explains the TE patterns. Chen et al. (2020) also found concordant TE changes in five *Gossypium* allopolyploid species and, most strikingly, they reported consistent movement of TE copies from the larger parental subgenome into the smaller parental subgenome, suggesting a homogenization of the initial genome-size difference.

The observed departure from expectation in genome sizes of *Dactylorhiza* allotetraploids is associated with moderate, apparently unstructured TE dynamics, in particular retroelements (table 2, fig. 4*a*, fig. 5; supplementary fig. S2, Supplementary Material online), but is mostly driven by amplification of a MITE-like element. It is noteworthy that this putative tandem repeat is found at such low copy-number in the larger paternal genome of *D. incarnata* (fig. 4*b* and table 2) that there is no detectable FISH signal in contrast to the maternal species (supplementary fig. S3*a*and*b*, Supplementary Material online). In the allopolyploids, clear amplification (and thus likely spread) of the MITE-like element occurs in both subgenomes (fig. 6; supplementary fig. S3*c*and*d*, Supplementary Material online), which, in addition to overexpression of the RdDp pathway and DNA integration in *D. incarnata* in comparison to *D. fuchsii* (Balao et al. 2017), could suggest that the silencing mechanisms of the smaller maternal genome may control activity of a large portion of TEs in the allopolyploids. However, it does not control that of the MITE-like element, which shows a high copy number already in the maternal genome. Tandem repeats are known to quickly evolve and in general not under the same constraints as other types of repeats. When it comes to tandem repeats and especially satellites, the “library hypothesis” (Ruiz-Ruano et al. 2016) proposes that a variety of satDNA families in an ancestral species can diverge and expand/retract rapidly between lineages (Koukalova et al. 2010; Garrido-Ramos 2017). This rapid rate of evolution can result in considerable differences in the satDNA landscape among closely related species (Koukalova et al. 2010; Ambrožová et al. 2011; Emadzade et al. 2014; Samoluk et al. 2017; Belyayev et al. 2020; Palacios-Gimenez et al. 2020) and among populations within species (Garrido-Ramos 2017).

### Evolutionary Dynamics of TEs Are Remarkably Consistent among Independently Formed Sibling Allopolyploids

It is widely accepted that most polyploids have multiple origins in time and place, creating an array of populations with distinct genetic, ecological, morphological, and physiological properties (Soltis and Soltis 1999, Paun et al. 2007). The sibling marsh orchids studied here show distinct ecological preferences and largely specific distributions, a legacy of their individual evolutionary histories (Hedrén 1996; Dijk and Grootjans 1998; Pillon et al. 2007; Paun et al. 2011, Wolfe et al. 2021). Here, we evaluated stochasticity of TE amplification after allopolyploidization and found little variation between sibling allopolyploids suggesting no significant “genomic shock” following their formation. In contrast, species-specific DNA methylation patterns have been previously documented in natural populations of *D. majalis* and *D. traunsteineri*, with almost 10% of the investigated cytosines found to be fully methylated in one polyploid and unmethylated in the other (Paun et al. 2010; Trucchi et al. 2016). This suggests that although the TE types and proportions over the entire genomes appear to be tightly regulated and consistent among sibling allopolyploids, there may be high variation among individual TE insertion sites. Individual TE insertions can trigger a shift in phenotype (Chuong et al. 2017; Dubin et al. 2018), potentially contributing molecular and ecological individuality to each of the sibling allopolyploids. Such locus-specific effects of TE insertions and their potential phenotypic effects remain to be investigated in future studies.

## Materials and Methods

### Plant Material, DNA, and Illumina Sequencing

Thirty-eight samples were included in the genomic analyses (supplementary table S3, Supplementary Material online) in addition to 46 other samples that have been used for genome-size estimations (supplementary table S1, Supplementary Material online). The sampling aimed to represent as much as possible the variation in each species, for example, by covering much of the distribution of each species. Total DNA was isolated from silica-dried leaves using a cetyl trimethylammonium bromide (CTAB) procedure (Doyle and Doyle 1990) or the DNeasy Plant Mini Kit (Qiagen, Venlo, Netherlands). DNA was purified with the Nucleospin gDNA clean-up kit (Macherey-Nagel, Düren, Germany) following the manufacturer’s protocol. The DNA was further sheared to an average 450 bp size using a Bioruptor Pico (Diagenode). Individually indexed high-throughput sequencing libraries were prepared using the TruSeq DNA PCR-Free Library Kit (Illumina Inc.). The Illumina HiSeq platform was used to generate paired-end 126 or 150 bp reads for each library (VBCF, NGS Unit, Vienna, Austria).

### Genome Size Estimation

Genomes size was estimated following Temsch et al. 2010. Young fruits and leaves were collected and stored at 4 °C for up to a week until used for flow cytometry. The material was co-chopped with a suitable internal standard (Galbraith et al. 1983) in cold Otto’s isolation buffer (Otto et al. 1981). *Solanum pseudocapsicum* (1.295 pg/1C; Temsch et al. 2010) or *Pisum sativum* (4.42 pg/1C; Greilhuber and Ebert 1994) were used as internal standards. The suspension was first filtered through a 30 μm nylon mesh and incubated together with RNase at 37 °C for 30 min. Then the suspension was stained with propidium iodide dissolved in Otto’s buffer II for about one hour at 4 °C. Genome size measurements were done on a CyFlow ML flow cytometer (Partec, Münster, Germany) equipped with a green laser (100 mW, 532 nm, Cobolt Samba; Cobolt AB, Stockholm). For each sample, an average of three runs with 3,333 particles was performed (supplementary table S1, Supplementary Material online); the results from all runs were averaged to estimate the final genome size. Multiple measurements were conducted to overcome potential biases in the genome size estimation (cf. Greilhuber et al. 2007).

As a reference point for what to expect by additivity of the parental genome sizes *in silico* allopolyploid genome size were computed by pairwise combining samples of the diploid parental species. To determine species pairs with significantly different genome sizes a pairwise *t*-test was performed with a Benjamini and Hochberg (1995) multiple testing correction.

### Data Preparation and Repeat Content Estimation

Raw Illumina sequence pairs were pre-processed for quality using a custom Python script (qualityFilterPairEnd.py available at https://github.com/mc-er/dact-TEs/), which removes read pairs with quality scores lower than 20 for a maximum of 5% of the total read length. Adapters were removed using the program cutadapt v.2.10 (Martin 2011) for Illumina TruSeq adapters, discarding any pairs with trimmed reads or those containing indeterminate bases (N). Following pre-processing, read pairs were trimmed to a length of 125 bp and mapped against the plastid genome of *D. fuchsii* (GenBank Accession number MK908418) using BWA (Li and Durbin 2009). Read pairs with high mapping scores towards the plastid genome were discarded to unambiguously focus the repeat content estimation solely on the nuclear genome.

RepeatExplorer v.2.3.7 (Novak et al. 2013; Novak et al. 2020b) was used to estimate repeat content of each parental species and the five sibling allopolyploids. To capture potential intraspecies variation, sequences from five individuals across the range of each species (supplementary table S3, Supplementary Material online) were used in the RepeatExplorer analyses (comparative analysis mode). First an analysis was run for each species (analyses 1–7; supplementary table S4, Supplementary Material online) using as many reads as possible with the maximum memory allowed (TAREAN_MAX_MEM = 110 Gb) to provide the basis of cluster annotation of the comparative analysis run with all species together. RepeatExplorer optimized memory usage and therefore the number of reads used per individual depending on the exactly-matching “repetitiveness” identified for each individual (Novak et al. 2020b). Lastly, a comparative analysis was conducted for parental diploids and allopolyploids together using a number of reads proportional to their genome sizes (analysis 8; supplementary table S4, Supplementary Material online). Prior to pooling species samples into a species representative for the comparative analyses, pairwise comparisons of cluster sizes were made between all samples within species in order to only include samples with similar repeat dynamics (supplementary figs. S5–S11, Supplementary Material online). All analyses used the Viridiplantae v.3.0 database (Neumann et al. 2019), automatic filtering for abundant satellites, keeping the read name and an “extra-long” analysis.

### Repeat Annotation

The RepeatExplorer clusters were first annotated based on superclusters. Each cluster within a supercluster was annotated according to the majority of matches found across all clusters to a TE protein-domain database (Neumann et al. 2019). Contigs from the remaining clusters for which no domains were present were inspected using clview and dotter v.4.44.1 (Sonnhamme and Durbin 1995) to detect insertion sites and inverted repeat signatures. Satellite DNAs were recovered using TAREAN, a module of the RepeatExplorer (Novak et al. 2017) and manually annotated using dotter v.4.44.1 (Sonnhammer and Durbin 1995). Clusters exclusively exhibiting insertion sites with no other characteristics typical of known repeats were left unclassified. The clusters returned by RepeatExplorer comparative analysis were annotated by cross referenced annotations from the species analysis with custom Python scripts (comparative_annotation.py and annotateCOMP.py available at https://github.com/mc-er/dact-TEs/). Clusters with ≥ 20% of reads with an annotation from the species analysis and the second major annotation being unclassified (> 50%) were given the annotation based on the species analyses.

### Parental Bias

The clusters identified in the comparative analysis (analysis 8; supplementary table S4, Supplementary Material online) were first filtered to only keep clusters with a proportion ≥ 0.001 out of analysed reads. The remaining clusters were then classified in two further steps. First, the clusters were regarded either as originally larger in one of the parental genomes or similar in size in the parents. For a cluster to be classified as originally larger in one of the parental genomes, the size difference between the parental clusters had to be ≥ 20%; if the difference was < 20% the cluster was classified as “similar” in size. The second classification compared the observed cluster size in the sibling allopolyploids to the expected value of an *in silico* allopolyploid. Clusters were retained and reported in fig. 5 if at least one of the sibling allopolyploids exceeded a threshold difference of ±20% to the expected *in silico* value.

### Chromosome Preparations

Root tips were harvested from cultivated plants, pre-treated with ice-cold water for 16 h, fixed in 3:1 ethanol:acetic acid fixative for 24 h at 4 °C and stored at −20 °C until further use. Selected root tips were rinsed twice for 5 min in distilled water, and twice for 5 min in citrate buffer (10 mM sodium citrate, pH 4.8). Then the root tips were digested in 0.3% cellulase, cytohelicase and pectolyase (all Sigma Aldrich) in citrate buffer at 37 °C for 3 h. After digestion, individual root tips were dissected on a microscope slide in 20 μL acetic acid and spread on the slide placed on a metal hot plate (50 °C) for *ca.* 30 s. This preparation was fixed in freshly prepared 3:1 ethanol:acetic acid fixative by dropping the fixative around the drop of acetic acid and into it. The preparation was dried using a hair dryer and staged using a phase contrast microscope. Chromosome preparations were treated with 100 μg·mL^−1^. RNase in 2 × sodium saline citrate (SSC; 20 × SSC: 3 M sodium chloride, 300 mM trisodium citrate, pH 7.0) for 60 min and with 0.1 mg·ml^−1^ pepsin in 0.01 M HCl at 37 °C for 5 min; then postfixed in 4% formaldehyde in 2 × SSC for 10 min, washed in 2 × SSC twice for 5 min, and dehydrated in an ethanol series (70, 90, and 100%, 2 min each).

### DNA Probes

A synthetic oligonucleotide probe was designed for the MITE-like repeats. A target sequence of 60 nt (AATTGCGAGTCGCATAGTTTAGGTAATATACGCAGAACACGCGCCCTTTGAAAATAGACG) was selected from DNA alignments using Geneious v.11.1.5 software package v.2 to minimize self-annealing and formation of hairpin structures. The DNA probe preparation and labelling followed published protocols (Mandáková and Lysak 2016). For GISH, total genomic DNA (gDNA) was extracted from young leaves of *D. incarnata* and *D. fuchsii* according to Dellaporta et al. (1983) followed by RNase treatment (50 μg·mL^−1^). Extracted gDNA was checked for protein, starch and RNA contamination using a Beckmann photospectrometer and ran on a 1% (w/v) agarose gel in 1 × Tris-acetate-EDTA (TAE) buffer. All DNA probes were labelled with biotin-dUTP or digoxigenin-dUTP by nick translation as described in Mandáková and Lysak (2016).

### In Situ Hybridization

Selected labelled DNA probes were pooled together, ethanol precipitated, dissolved in a 20 μL mixture containing 50% formamide, 10% dextran sulphate and 2 × SSC, and pipetted onto each of the microscopic slides. The slides were heated at 80 °C for 2 min and incubated at 37 °C overnight. The hybridized probes were visualized through fluorescently labeled antibodies against biotin-dUTP (red) and digoxigenin-dUTP (green) as in Mandáková and Lysak (2016). Chromosomes were counterstained with 4′,6-diamidino-2-phenylindole (DAPI, 2 μg·mL^−1^) in Vectashield antifade. Fluorescence signals were analysed and photographed using a Zeiss Axioimager epifluorescence microscope and a CoolCube camera (MetaSys- tems, Altlussheim, Germany). Individual images were merged and processed using the Photoshop CS software (Adobe Systems).

## Supplementary Material

msac167_Supplementary_Data

## Data Availability

The raw Illumina sequencing data underlying this article are available in NCBI SRA database at https://www.ncbi.nlm.nih.gov/sra under the BioProject PRJNA845973 (accession numbers SRX15595094-SRX15595128).
