# Supplementary material for: Repeat Dynamics across Timescales: A Perspective from Sibling Allotetraploid Marsh Orchids (Dactylorhiza majalis s.l.)
Source: Mol Biol Evol. 2022 Jul 29;39(8):msac167. doi: 10.1093/molbev/msac167 (PMC9366187; doi:10.1093/molbev/msac167)
Supplement: msac167_Supplementary_Data [file msac167_supplementary_data.pdf]

Supplementary Figures and Supplementary Tables

Eriksson et al.

Repeat dynamics across timescales: a perspective from sibling allotetraploid marsh orchids (*Dactylorhiza majalis* s.l.)

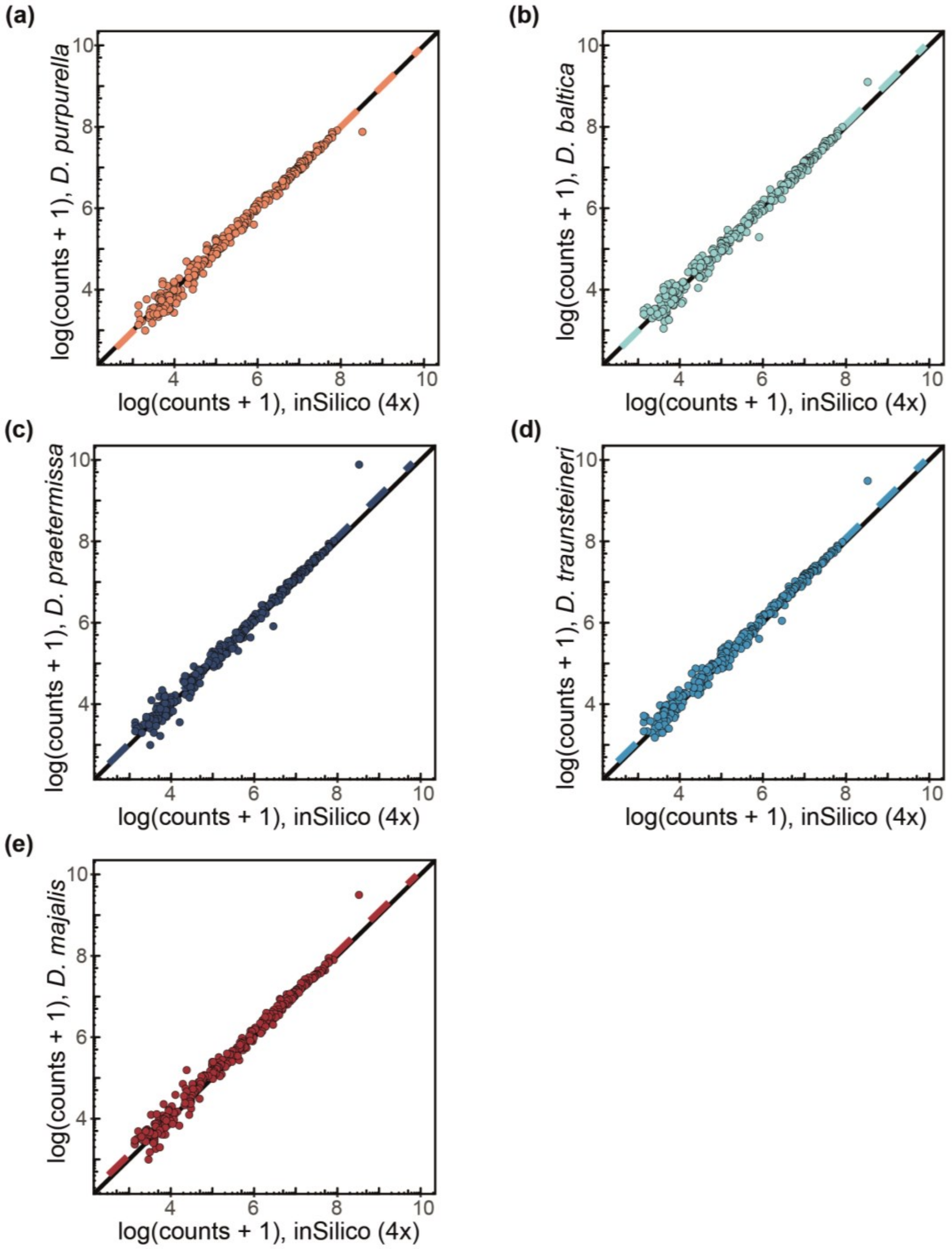

Supplementary Figure S1. Repeat comparison for each sibling allotetraploid (Y-axis) against an additive *in silico* allopolyploid (X-axis). The solid black diagonal line represents observed = expected, and the coloured dashed line the trend. (***a***) *D. purpurella.* (***b***) *D. baltica*. (***c***) *D. praetermissa*. (***d***) *D. traunsteineri*. (***e***) *D. majalis*.

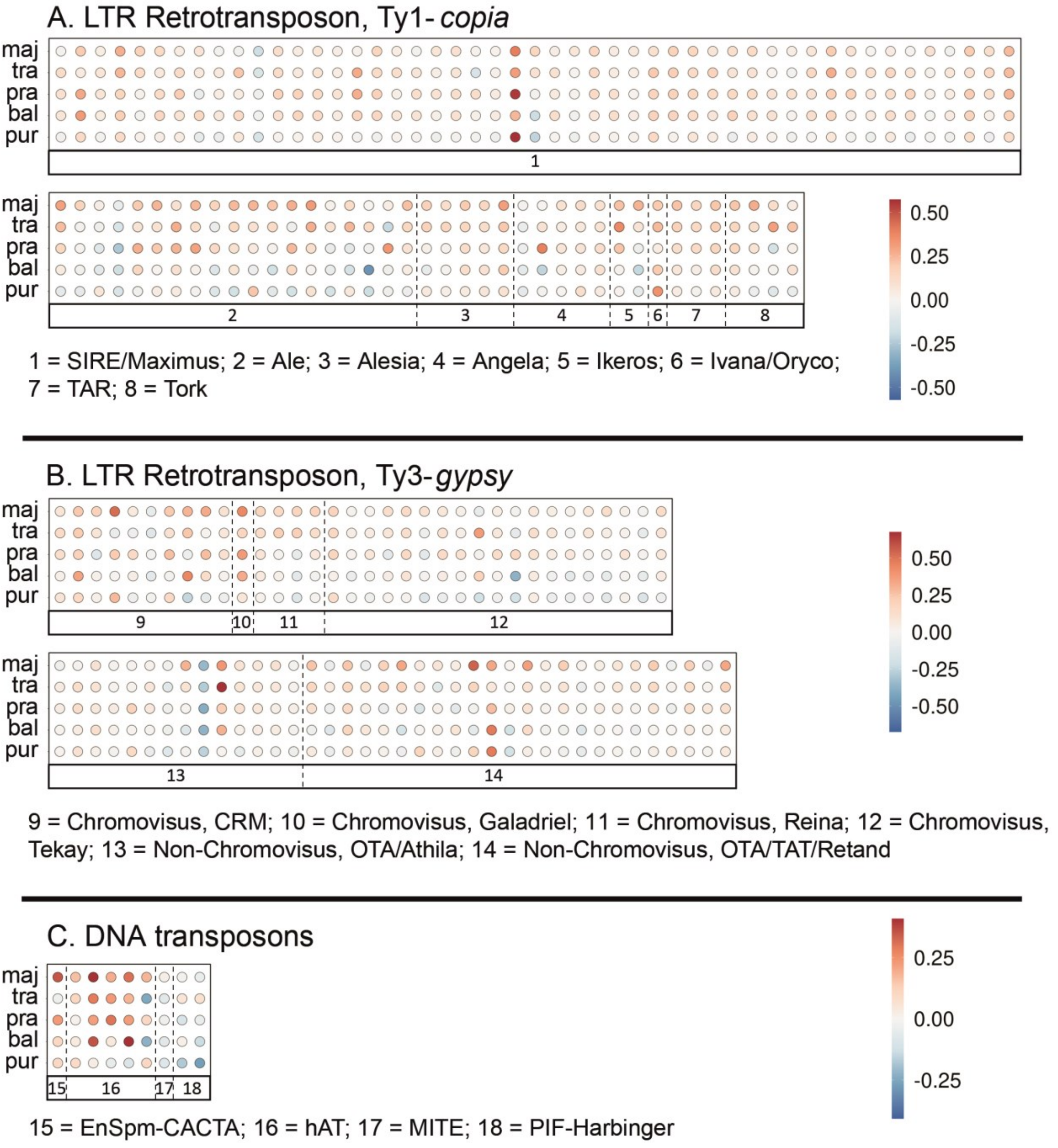

Supplementary Figure S2. Cluster specific effects between each sibling allotetraploids. Normalised cluster changes for families of LTR retrotransposons, Ty1-*copia* (***a***), LTR retrotransposons, Ty3-*gypsy* (***b***), and DNA-transposons (***c***). Red tones, larger than expected by parental additivity. Blue tones, less than expected by parental additivity. Normalisation applied:

$$tan\left(\left(\frac{obs.counts}{exp.counts}\right)-1\right).$$

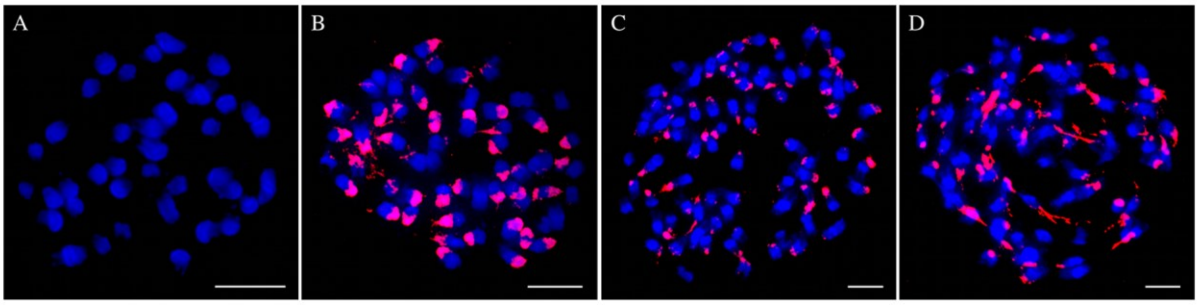

Supplementary Figure S3. FISH localization of MITE2 repeat (purple fluorescence) on mitotic metaphase chromosomes of *D. incarnata* (***a***), *D. fuchsii* (***b***), *D. majalis* (***c***), and *D. traunsteineri* (***d***). Chromosomes were counterstained by DAPI. Scale bars, 10 µm.

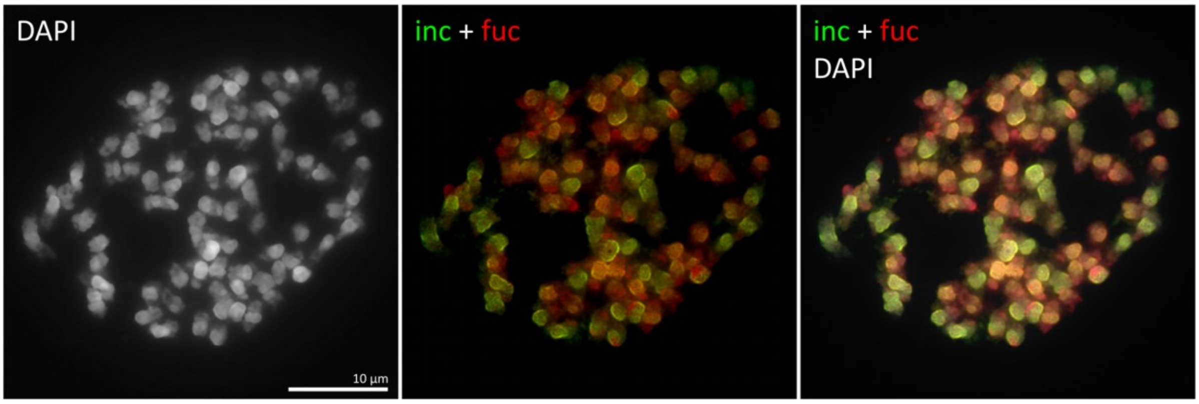

Supplementary Figure S4. GISH in *D. traunsteineri* with genomic DNA of *D. incarnata* and *D. fuchsii* as probes. Chromosomes were counterstained by DAPI; GISH signals are shown in colour as indicated. Scale bar, 10 µm.

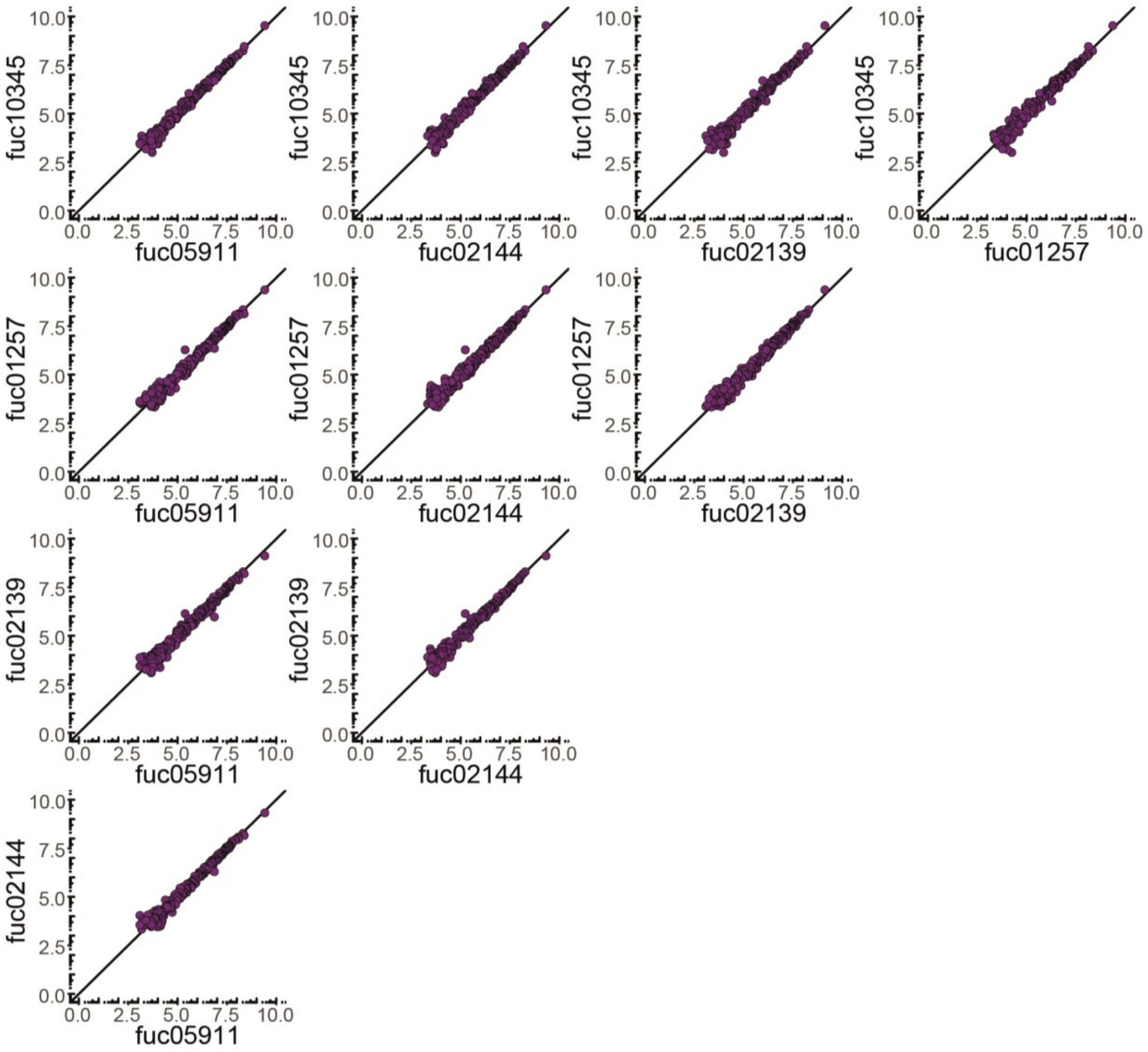

Supplementary Figure S5. Pairwise sample comparison of repeat clusters for *D. fuchsii* to investigate within species variation and exclude potential variable samples from the species pool in the comparative analysis. Read counts are normalised by log(counts+1) for better visualisation.

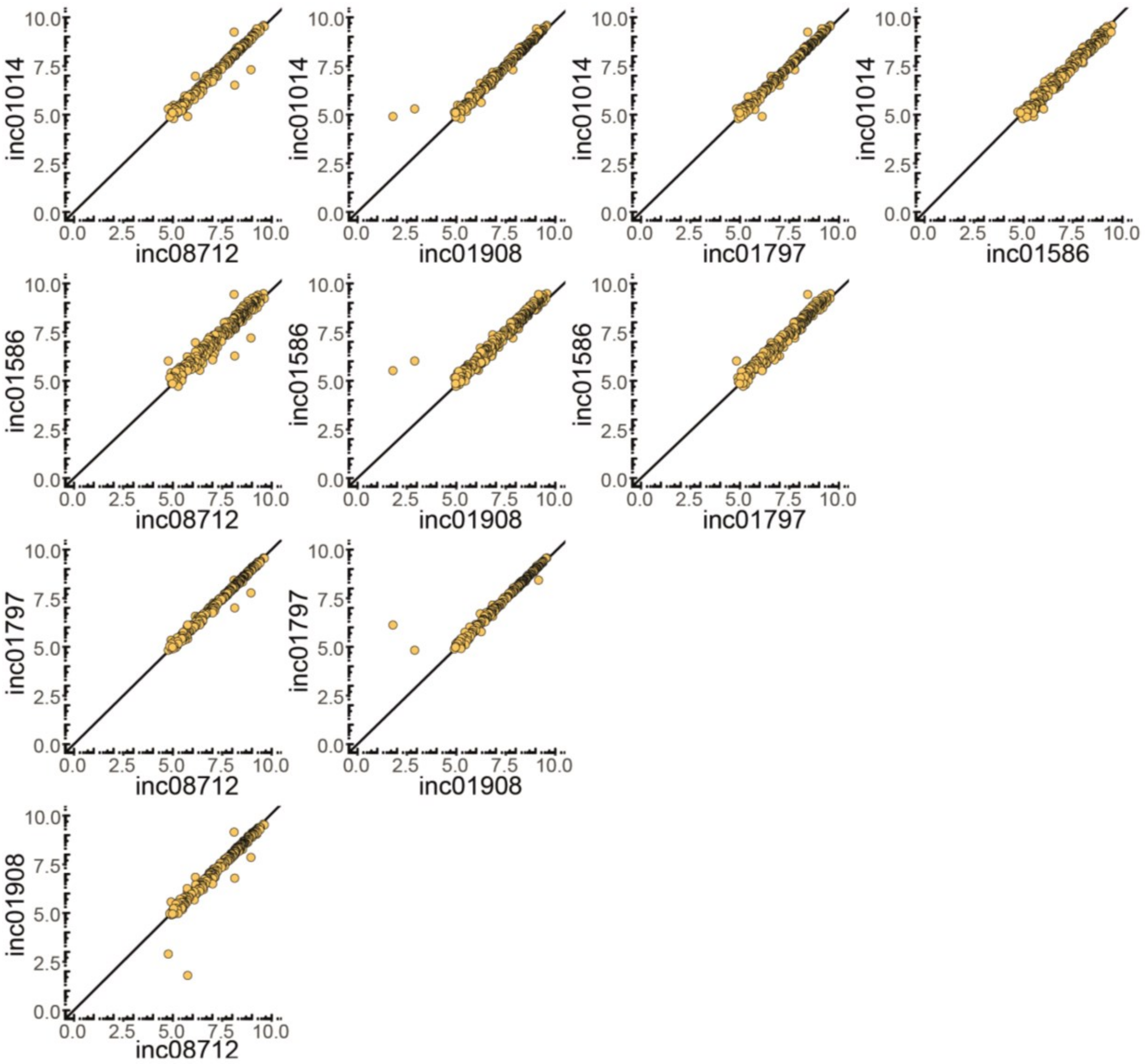

Supplementary Figure S6. Pairwise sample comparison of repeat clusters for *D. incarnata* to investigate within species variation and exclude potential variable samples from the species pool in the comparative analysis. Read counts are normalised by log(counts+1) for better visualisation.

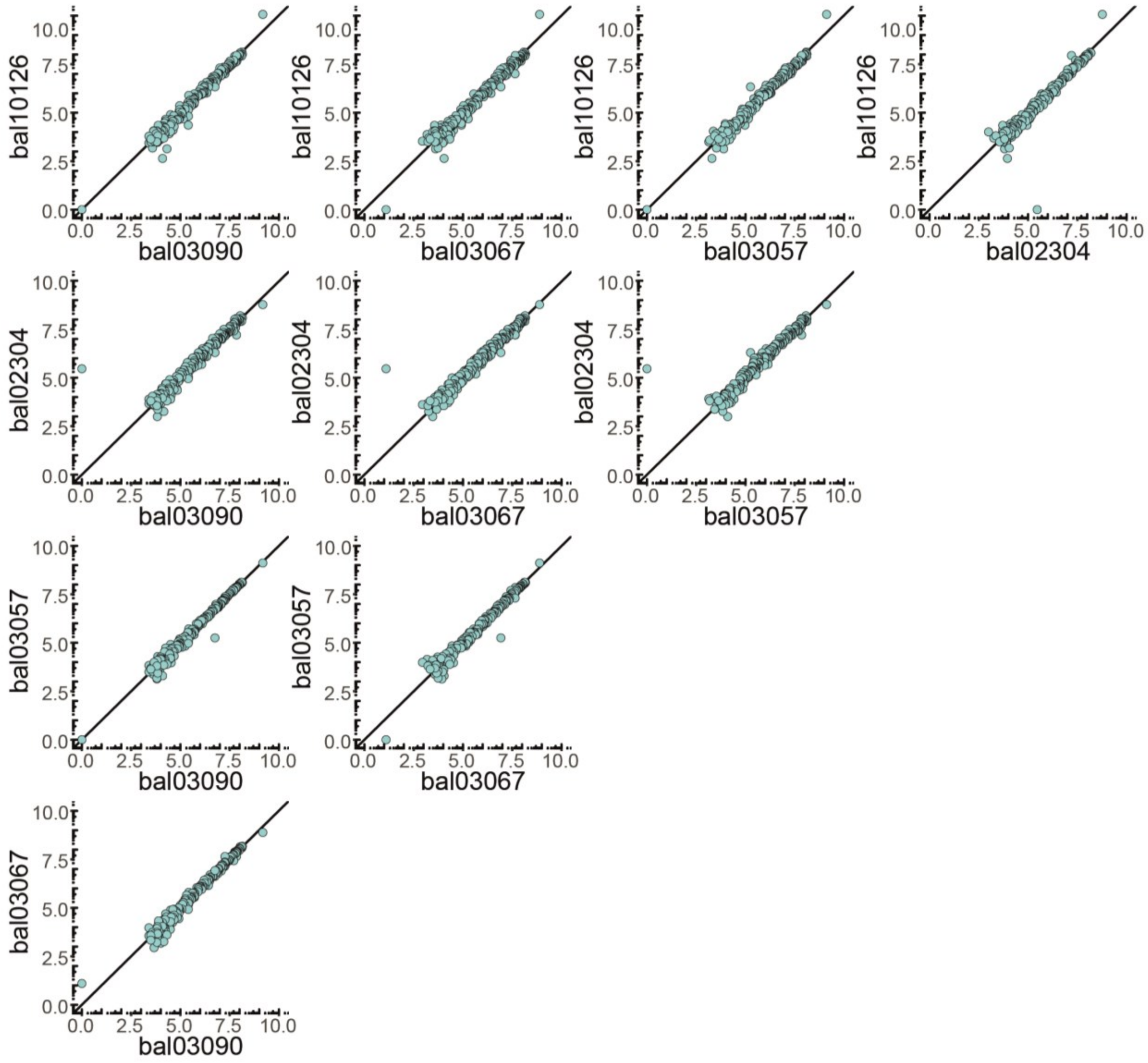

Supplementary Figure S7. Pairwise sample comparison of repeat clusters for *D. baltica* to investigate within species variation and exclude potential variable samples from the species pool in the comparative analysis. Read counts are normalised by log(counts+1) for better visualisation.

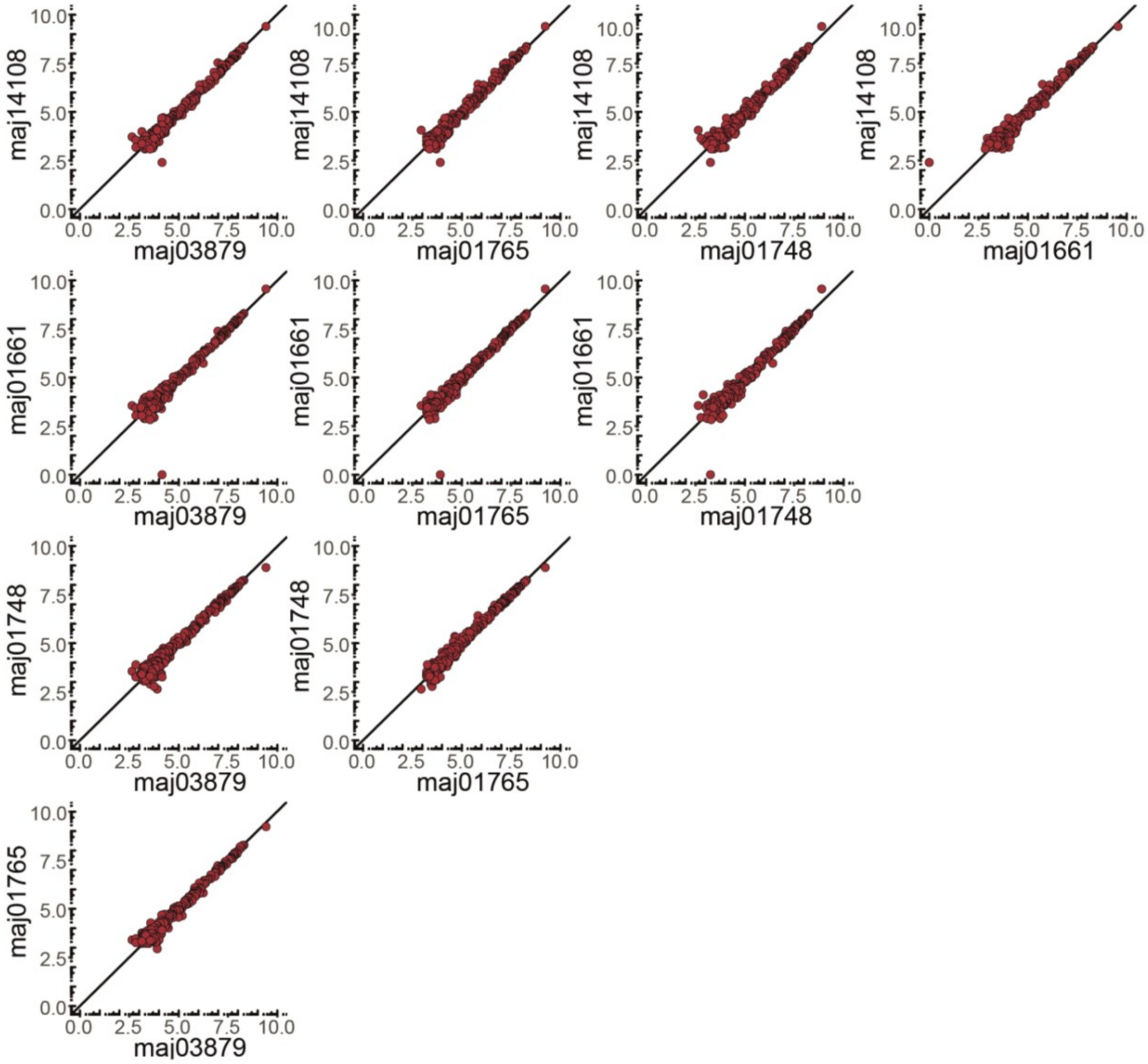

Supplementary Figure S8. Pairwise sample comparison of repeat clusters for *D. majalis* to investigate within species variation and exclude potential variable samples from the species pool in the comparative analysis. Read counts are normalised by log(counts+1) for better visualisation.

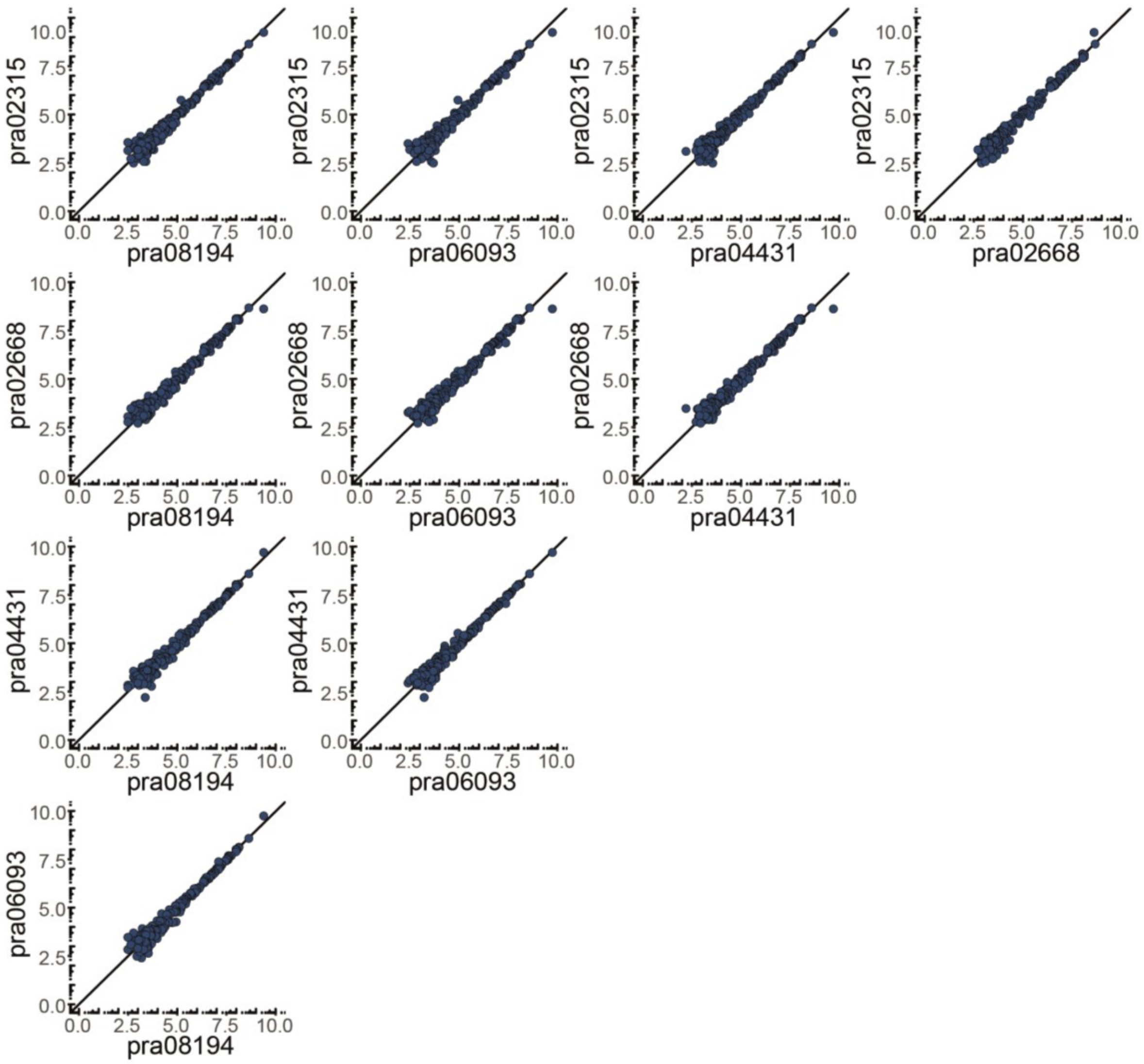

Supplementary Figure S9. Pairwise sample comparison of repeat clusters for *D. pratermissa* to investigate within species variation and exclude potential variable samples from the species pool in the comparative analysis. Read counts are normalised by log(counts+1) for better visualisation.

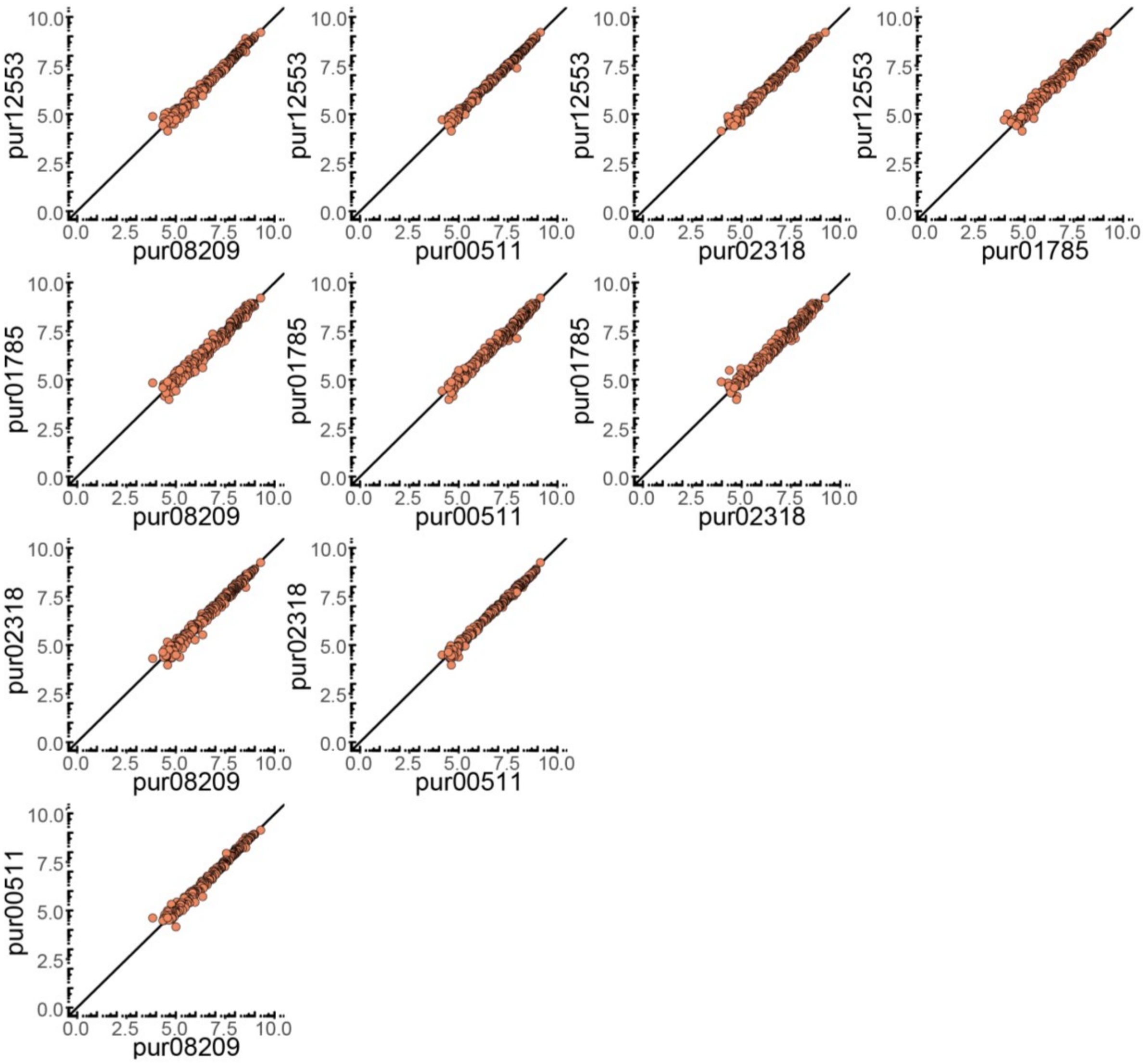

Supplementary Figure S10. Pairwise sample comparison of repeat clusters for *D. purpurella* to investigate within species variation and exclude potential variable samples from the species pool in the comparative analysis. Read counts are normalised by log(counts+1) for better visualisation.

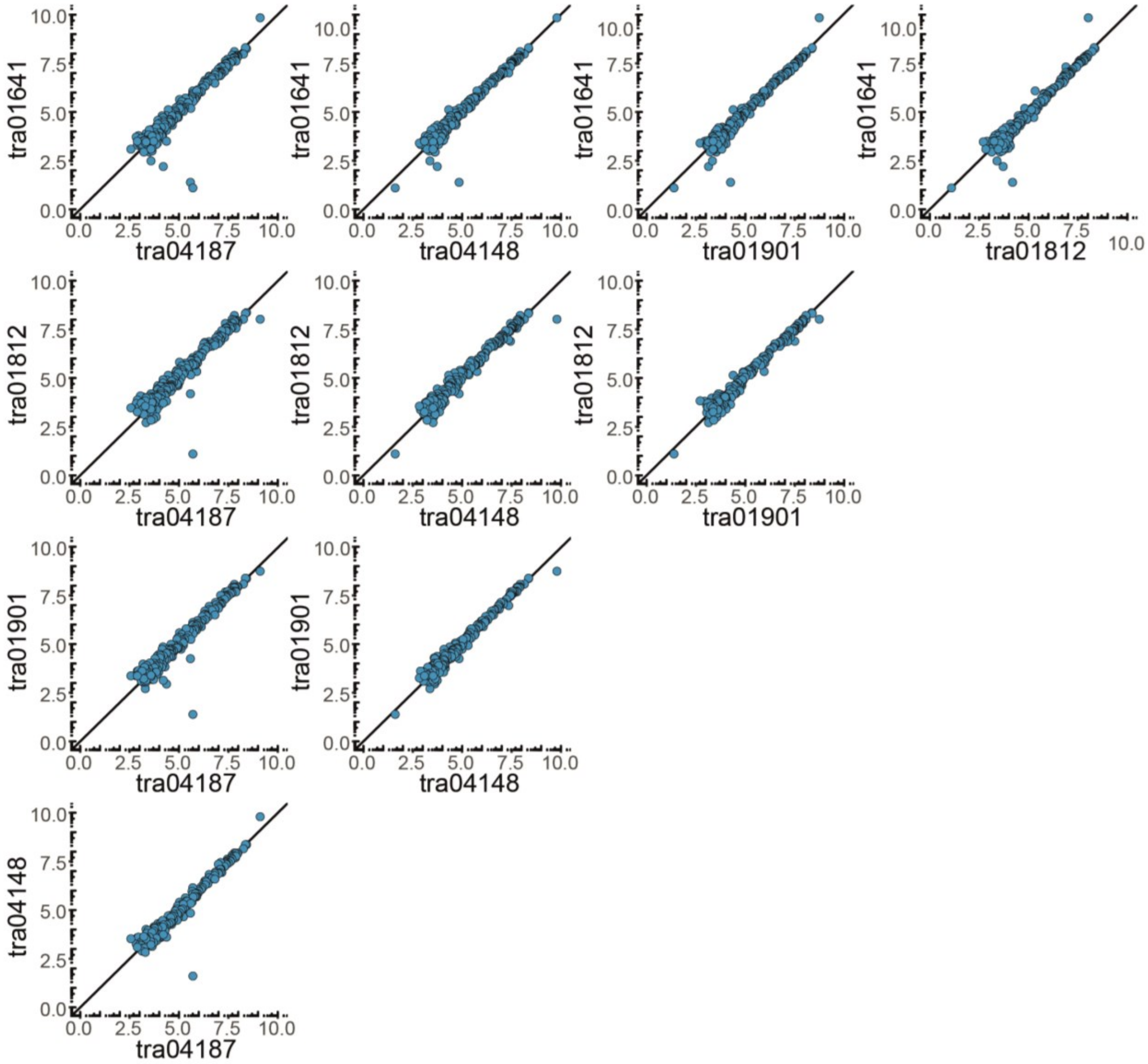

Supplementary Figure S11. Pairwise sample comparison of repeat clusters for *D. traunsteineri* to investigate within species variation and exclude potential variable samples from the species pool in the comparative analysis. Read counts are normalised by log(counts+1) for better visualisation.

Supplementary Table S1. Measurements of genome size for each sample. Collectors: MC - Mark W. Chase; MH - Mikael Hedrén; OP - Ovidiu Paun; RB - Richard M. Bateman, KT - Kadri Tali.

Species	Sample	Ploidy	Collector	Country	N	Mean 1C (pg)	SEM
D. fuchsii	fucBRI5	2x	OP	BRI	5	2.76	0.15
D. fuchsii	fucALP15	2x	OP	AUT	7	2.93	0.09
D. fuchsii	fucNS	2x	OP	AUT	3	3.15	0.01
D. fuchsii	fucSW	2x	OP	SWE	2	2.79	0.20
D. incarnata	incPYR1	2x	OP	FRA	7	3.70	0.03
D. incarnata	incPYR3	2x	OP	FRA	3	3.45	0.02
D. incarnata	inc1ALP13	2x	OP	AUT	2	3.35	0.05
D. incarnata	inc2ALP13	2x	OP	AUT	4	3.66	0.03
D. incarnata	incALP11	2x	OP	AUT	4	3.71	0.03
D. incarnata	incBRI5	2x	OP	BRI	6	3.68	0.01
D. incarnata	incSWE	2x	OP	SWE	5	3.64	0.01
D. baltica	bal2303	4x	OP	POL	3	6.83	0.00
D. baltica	bal2305	4x	OP	POL	3	6.82	0.01
D. baltica	bal2307	4x	OP	POL	3	6.83	0.04
D. baltica	bal2308	4x	OP	POL	3	6.82	0.03
D. baltica	bal2319	4x	KT	EST	3	6.80	0.00
D. baltica	bal2320	4x	KT	EST	3	6.90	0.02
D. baltica	bal2321	4x	KT	EST	3	6.84	0.00
D. majalis	majALPx	4x	OP	AUT	4	6.98	0.02
D. majalis	majALP13	4x	OP	AUT	2	7.50	0.00
D. majalis	majALP9	4x	OP	AUT	4	7.38	0.11
D. majalis	maj1ALP15	4x	OP	AUT	1	7.28	-
D. majalis	maj2ALP15	4x	OP	AUT	3	7.23	0.02
D. majalis	majALP10	4x	OP	AUT	12	7.35	0.08
D. majalis	majPYR1	4x	OP	AUT	7	7.40	0.06
D. majalis	majSWE	4x	OP	SWE	11	7.15	0.05
D. praetermissa	pra2310	4x	RB	BRI	3	7.79	0.01
D. praetermissa	pra2311	4x	RB	BRI	1	7.63	-
D. praetermissa	pra2315	4x	RB	BRI	3	7.12	0.02
D. praetermissa	pra2316	4x	RB	BRI	3	7.18	0.01
D. praetermissa	pra2317	4x	MC	BRI	1	7.30	-
D. purpurella	pur2318	4x	MC	BRI	4	6.74	0.06
D. purpurella	pur2327	4x	RB	BRI	1	6.73	-
D. purpurella	pur2328	4x	RB	BRI	1	6.64	-
D. purpurella	pur2329	4x	RB	BRI	1	6.65	-
D. purpurella	pur2330	4x	RB	BRI	3	6.67	0.02
D. traunsteineri	traBRI1	4x	OP	BRI	5	7.23	0.03
D. traunsteineri	traBRI2	4x	OP	BRI	5	7.25	0.01
D. traunsteineri	traALP9	4x	OP	AUT	2	7.38	0.02
D. traunsteineri	traALP13	4x	OP	AUT	3	7.57	0.01
D. traunsteineri	traSWE	4x	OP	SWE	15	6.83	0.14
D. traunsteineri	traALP8	4x	OP	AUT	2	7.18	0.04

Supplementary Table S2. Results of statistical analysis of genome size performed in R version 4.2.0. Pairwise t-test with correcting for multiple testing using Benjamini & Hochberg (1995). InSilico, additive expectation relative to the diploid parents; pur, *D. purpurella*; bal, *D. baltica*; pra, *D. praetermissa*; tra, *D. traunsteineri*; maj, *D. majalis*.

	inSilico	pur	bal	pra	tra	maj
**pur**	0.08283	-				
**bal**	0.00031	0.2262	-			
**pra**	2.90E-12	1.10E-06	1.50E-05	-		
**tra**	1.10E-10	3.60E-05	0.00062	0.20904	-	
**maj**	1.10E-12	4.40E-06	7.30E-05	0.30039	0.67701	-

Supplementary Table S3. Genomic abundance of repeats in Mbp for each species, calculated from 2C genome sizes. Species abbreviations are explained in the legend of Supplementary Table S2.

Repeat type/family	fuc	inc	pur	bal	pra	tra	maj
LTR Retrotransposons	2567.0	3491.8	6309.4	6283.9	6612.8	6499.4	6550.4
Ty1-copia	1312.2	1950.1	3429.6	3476.2	3588.6	3540.0	3531.5
SIRE/Maximus	974.1	1492.5	2578.7	2647.3	2720.4	2647.9	2620.2
Other	338.1	464.6	850.9	828.9	868.2	892.1	925.6
Ty3-gypsy	1002.8	1161.6	2199.1	2152.6	2315.2	2279.8	2306.9
Chromovirus	464.1	387.2	863.9	842.3	926.1	892.1	911.4
CRM	80.2	49.3	130.9	133.7	144.7	127.4	142.4
Tekay	361.0	316.8	680.7	668.5	738.0	708.0	712.0
Other	22.9	21.1	52.4	53.5	57.9	56.6	57.0
Non-chromovirus	538.6	774.4	1348.3	1310.3	1389.1	1387.7	1395.5
Athila	183.4	323.8	523.6	521.4	549.9	538.1	526.9
Retand	355.3	450.6	824.7	788.8	839.3	849.6	868.6
LTR Unclassified	252.1	380.2	680.7	668.5	709.0	693.8	712.0
Non-LTR retrotransposons	22.9	28.2	52.4	53.5	57.9	56.6	57.0
LINE	22.9	28.2	52.4	53.5	57.9	56.6	57.0
DNA transposons	22.9	28.2	52.4	53.5	57.9	56.6	57.0
hAT	17.2	21.1	39.3	40.1	28.9	42.5	42.7
MITE	3.4	4.2	7.9	6.7	7.2	7.1	8.5
Other	5.7	7.0	13.1	13.4	14.5	14.2	14.2
Tandem repeats	298.0	133.8	327.3	615.0	1143.1	821.3	825.9
rDNA	22.9	28.2	39.3	40.1	28.9	28.3	42.7
satDNA	275.0	105.6	288.0	574.9	1114.2	793.0	783.2
MITE-like	206.3	35.2	130.9	427.8	955.0	637.2	640.8
Unclassified	418.3	605.4	1086.5	1069.6	1128.7	1118.6	1153.4
Total TEs	2612.9	3548.2	6401.0	6390.9	6728.6	6612.7	6678.6
Total repeats	4068.3	5195.5	9437.9	9800.2	10678.9	10336.8	10338.2

Supplementary Table S4. Samples used for the repeat analysis and the number of reads at different stages of the analysis. Collectors: DS - David Ståhlberg, EW - Erik Westberg, HP - Henrik Ærenlund Pedersen, MC - Mark W. Chase; MH - Mikael Hedrén; OP - Ovidiu Paun; RB - Richard M. Bateman, SN - Sofie Nordström, SS - Sebastian Sczepanski.

Species	Sample ID	Collector	Country	Lat.	Long.	Reads Sequenced	Filtered reads	Reads in species analysis 1-7	Reads in comparative analysis 8
D. fuchsii	1257	OP	BRI	54.283	-0.683	6,735,326	5 814 229	323,780	
D. fuchsii	2139	OP	SWE	60.617	17.617	20,103,228	4 047 820	323,698	
D. fuchsii	2144	OP	AUT	47.700	15.200	21,682,260	4 440 354	323,918	118,958
D. fuchsii	5911	DS	RUS	66.783	30.150	17,305,004	3 659 598	323,696	
D. fuchsii	10345	MH	FRA	46.858	1.290	26,385,264	5 991 410	324,070	
**Analysis 1, total:**								**1,619,162**	
D. incarnata	1014	OP	FRA	42.862	1.981	33,110,772	6 532 268	1,409,522	
D. incarnata	1586	OP	AUT	47.283	11.183	18,788,436	3 538 428	1,409,472	
D. incarnata	1797	OP	BRI	54.667	-2.250	11,921,652	10 262 301	1,409,276	145,054
D. incarnata	1908	OP	SWE	57.817	18.883	21,812,480	4 351 815	1,408,248	
D. incarnata	8712	MH	IRL	53.373	-6.144	26,867,336	5 990 211	1,411,066	
**Analysis 2, total:**								**7,047,584**	
D. baltica	2304	OP	POL	54.233	22.843	4,874,212	4 217 397	346,114	
D. baltica	3057	SN	EST	58.333	21.967	10,788,049	9 502 572	345,950	
D. baltica	3067	DS	EST	57.717	26.503	5,143,011	4 496 282	346,342	278,331
D. baltica	3090	DS	EST	58.800	22.833	9,633,919	8 500 148	346,054	
D. baltica	10126	SS	GER	54.134	13.769	18,019,340	4 036 329	346,588	
**Analysis 3, total:**								**1,731,048**	
D. majalis	1661	OP	AUT	47.529	12.579	21,786,920	4 646 798	247,042	
D. majalis	1748	OP	FRA	42.859	0.495	11,656,812	2 366 655	246,804	
D. majalis	1765	OP	SWE	55.818	12.946	15,508,896	3 029 333	247,688	294,786
D. majalis	3879	EW	GER	53.434	10.104	19,964,412	4 090 828	247,166	
D. majalis	14108	MH	BEL	50.176	5.050	18,455,772	4 134 965	247,914	
**Analysis 4, total:**								**1,236,614**	
D. praetermissa	2315	RB	BRI	51.477	-0.297	10,560,758	9 235 857	183,872	
D. praetermissa	2668	MH	DEN	56.867	9.400	9,304,084	7 746 323	183,388	297,303
D. praetermissa	4431	SN	BRI	52.296	-0.416	5,562,390	4 806 891	183,736	

D. praetermissa	6093	MH	BRI	50.967	-1.333	8,058,631	6 955 646	183,090	
D. praetermissa	8194	HP	DEN	55.616	12.433	10,994,077	9 770 898	183,576	
**Analysis 5, total:**								**917,662**	
D. purpurella	511	MH	BRI	52.950	-4.083	11,543,909	10 180 118	881,366	
D. purpurella	1785	OP	BRI	54.783	-2.417	5,113,835	4 095 403	881,284	
D. purpurella	2318	MC	BRI	54.929	-2.954	7,317,242	6 461 438	879,278	259,753
D. purpurella	8209	HP	DEN	55.509	9.906	11,074,821	9 594 236	881,862	
D. purpurella	12553	MH	NOR	58.733	5.517	31,168,244	7 005 495	880,658	
**Analysis 6, total:**								**4,404,448**	
D. traunsteineri	1641	OP	AUT	47.450	12.367	19,033,808	4 073 281	245,232	
D. traunsteineri	1812	OP	BRI	57.417	-5.817	52,202,584	11 315 476	244,786	
D. traunsteineri	1901	OP	SWE	57.340	18.321	17,402,168	3 691 736	243,990	294,693
D. traunsteineri	4148	SN	FIN	67.567	26.867	13,498,084	2 974 119	245,014	
D. traunsteineri	4187	SN	EST	58.283	22.133	13,730,396	2 809 426	245,372	
**Analysis 7 & 8, total:**								**1,224,394**	**1,688,878**
